# Genome-wide study of resistant hypertension identified from electronic health records

**DOI:** 10.1371/journal.pone.0171745

**Published:** 2017-02-21

**Authors:** Logan Dumitrescu, Marylyn D. Ritchie, Joshua C. Denny, Nihal M. El Rouby, Caitrin W. McDonough, Yuki Bradford, Andrea H. Ramirez, Suzette J. Bielinski, Melissa A. Basford, High Seng Chai, Peggy Peissig, David Carrell, Jyotishman Pathak, Luke V. Rasmussen, Xiaoming Wang, Jennifer A. Pacheco, Abel N. Kho, M. Geoffrey Hayes, Martha Matsumoto, Maureen E. Smith, Rongling Li, Rhonda M. Cooper-DeHoff, Iftikhar J. Kullo, Christopher G. Chute, Rex L. Chisholm, Gail P. Jarvik, Eric B. Larson, David Carey, Catherine A. McCarty, Marc S. Williams, Dan M. Roden, Erwin Bottinger, Julie A. Johnson, Mariza de Andrade, Dana C. Crawford

**Affiliations:** 1 Department of Molecular Physiology and Biophysics, Vanderbilt University School of Medicine, Nashville, Tennessee, United States of America; 2 Biomedical and Translational Informatics, Geisinger Health System, Danville, Pennsylvania, United States of America; 3 Department of Biomedical Informatics, Vanderbilt University, Nashville, Tennessee, United States of America; 4 Department of Medicine, Vanderbilt University, Nashville, Tennessee, United States of America; 5 Department of Pharmacotherapy and Translational Research and Center for Pharmacogenomics, College of Pharmacy, University of Florida, Gainesville, Florida, United States of America; 6 Division of Epidemiology, Mayo Clinic, Rochester, Minnesota, United States of America; 7 Office of Research, Vanderbilt University, Nashville, Tennessee, United States of America; 8 Division of Biomedical Statistics and Informatics, Department of Health Sciences Research, Mayo Clinic, Rochester, Minnesota, United States of America; 9 Biomedical Informatics Research Center, Marshfield Clinic Research Foundation, Marshfield, Wisconsin, United States of America; 10 Group Health Research Institute, Seattle, Washington, United States of America; 11 Department of Preventive Medicine, Division of Health and Biomedical Informatics, Northwestern University, Chicago, Illinois, United States of America; 12 Center for Genetic Medicine, Northwestern University, Chicago, Illinois, United States of America; 13 Department Medicine, Feinberg School of Medicine, Northwestern University, Chicago, Illinois, United States of America; 14 Division of Genomic Medicine, National Human Genome Research Institute, Bethesda, Maryland, United States of America; 15 Epidemiology and Biostatistics, Institute for Computational Biology, Case Western Reserve University, Cleveland, Ohio, United States of America; 16 Department of Cardiovascular Diseases, Mayo Clinic, Rochester, Minnesota, United States of America; 17 Division of General Internal Medicine, Johns Hopkins University, Baltimore, Maryland, United States of America; 18 Department of Medicine, University of Washington Medical Center, Seattle, Washington, United States of America; 19 Weis Center for Research, Geisinger Health System, Danville, Pennsylvania, United States of America; 20 Essentia Institute of Rural Health, Duluth, Minnesota, United States of America; 21 Genomic Medicine Institute, Geisinger Health System, Danville, Pennsylvania, United States of America; 22 Department of Pharmacology, Vanderbilt University, Nashville, Tennessee, United States of America; 23 Charles R. Bronfman Institute for Personalized Medicine, Mount Sinai, New York, New York, United States of America; 24 Division of Cardiovascular Medicine, College of Medicine, University of Florida, Gainesville, Florida, United States of America; Ohio State University Wexner Medical Center, UNITED STATES

## Abstract

Resistant hypertension is defined as high blood pressure that remains above treatment goals in spite of the concurrent use of three antihypertensive agents from different classes. Despite the important health consequences of resistant hypertension, few studies of resistant hypertension have been conducted. To perform a genome-wide association study for resistant hypertension, we defined and identified cases of resistant hypertension and hypertensives with treated, controlled hypertension among >47,500 adults residing in the US linked to electronic health records (EHRs) and genotyped as part of the electronic MEdical Records & GEnomics (eMERGE) Network. Electronic selection logic using billing codes, laboratory values, text queries, and medication records was used to identify resistant hypertension cases and controls at each site, and a total of 3,006 cases of resistant hypertension and 876 controlled hypertensives were identified among eMERGE Phase I and II sites. After imputation and quality control, a total of 2,530,150 SNPs were tested for an association among 2,830 multi-ethnic cases of resistant hypertension and 876 controlled hypertensives. No test of association was genome-wide significant in the full dataset or in the dataset limited to European American cases (n = 1,719) and controls (n = 708). The most significant finding was *CLNK* rs13144136 at p = 1.00x10^-6^ (odds ratio = 0.68; 95% CI = 0.58–0.80) in the full dataset with similar results in the European American only dataset. We also examined whether SNPs known to influence blood pressure or hypertension also influenced resistant hypertension. None was significant after correction for multiple testing. These data highlight both the difficulties and the potential utility of EHR-linked genomic data to study clinically-relevant traits such as resistant hypertension.

## Introduction

Hypertension, or high blood pressure, affects nearly a third of the US adult population and is a major risk factor for coronary heart disease, stroke, and kidney disease [1, 2]. Pharmacological treatment of hypertension has been shown to lower blood pressure and substantially reduce the risk of coronary heart disease and stroke [3, 4]. While the number of patients tested and receiving treatment for hypertension has increased substantially over the last 40 years, a majority (~50%) of hypertensive Americans’ blood pressure remains uncontrolled [1, 5, 6]. Resistant hypertension is defined as blood pressure that remains ≥140/90 mm Hg despite use of three concurrent antihypertensive agents from different classes, one of which includes a diuretic [7]. Other definitions also include individuals who require four or more medications to achieve a blood pressure <140/90 mm Hg. The prevalence of resistant hypertension is progressively increasing and is currently estimated to affect 8–12% of adults with hypertension [8].

Along with age and obesity, well-established independent risk factors of resistant hypertension, several other lifestyle and biological risk factors are believed to contribute to resistant hypertension, including excess alcohol use, increased dietary sodium intake, and use of several classes of medications (such as non-steroidal anti-inflammatory drugs, corticosteroids, and calcineurin inhibitors) [9]. Genetic factors may also play a role [10]. Numerous genetic variants have been identified in studies of the genetic architecture of hypertension and variation in blood pressure [11, 12]. Additionally, hypertension pharmacogenomics research has implicated genes important in the inter-individual variability of response to specific antihypertensive drugs [13–15]. However, due to the small sample sizes, genetic studies of resistant hypertension have been limited in power and in scope (i.e., candidate gene studies) [16–21].

To help overcome the obstacle of limited sample size, we used data available from seven electronic health record (EHR) systems to identify both resistant hypertension cases and hypertensive individuals who responded well to a single antihypertensive medication (controlled hypertensives) in the electronic MEdical Records & GEnomics (eMERGE) Network [22, 23]. We then performed a genome-wide association study (GWAS) to identify common variants associated with resistant hypertension in 2,830 cases and 876 controls. We also assessed whether SNPs previously associated with blood pressure or hypertension in the literature were associated with resistant hypertension, a complex and clinically-relevant phenotype.

## Materials and methods

### eMERGE Network

The eMERGE Network and its studies are approved by the Institutional Review Board at each study site, which include Geisinger Health System, Group Health, Marshfield Clinic, Mayo Clinic, Mount Sinai School of Medicine, Northwestern University, University of Washington, Vanderbilt University [22, 23]. Participants at all study sites except for Vanderbilt University provided written, informed consent. Vanderbilt University’s biobank BioVU followed an opt-out model where DNA was extracted from discarded blood extracted for clinical purposes. The DNA linked to de-identified EHRs is considered “non-human subjects” as no personal identifying information is available to the investigators (The Code of Federal Regulations, 45 CFR 46.102 (f)) [24, 25].

**Fig 1:**
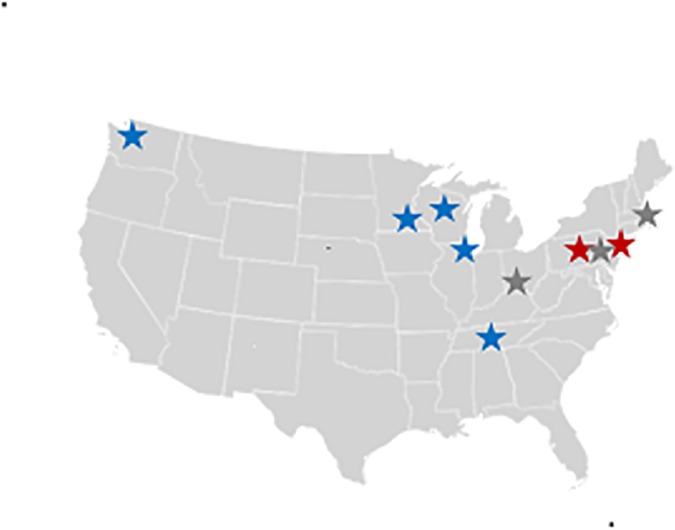
Resistant hypertension genome-wide association study in the eMERGE Network. The eMERGE Network conducted a genome-wide association study for resistant hypertension among adults drawn from the two funding phases of the network. The eMERGE Network I study sites that contributed data are denoted in blue and include Group Health/University of Washington in Seattle, WA; Marshfield Clinic in Marshfield, WI; Mayo Clinic in Rochester, MN; Northwestern University in Chicago, IL; and Vanderbilt University in Nashville, TN. The eMERGE Network II study sites that contributed data are denoted in red and include Geisinger Health System in Danville, PA and Mount Sinai School of Medicine in Manhattan, NY. Denoted in gray are eMERGE Phase II pediatric study sites not included in the present study (Boston Children’s Hospital in Boston, MA; Children’s Hospital of Philadelphia in Philadelphia, PA; and Cincinnati Children’s Hospital in Cincinnati, OH).

The eMERGE Network, initially funded in 2007 by the National Human Genome Research Institute (NHGRI), consisted of five EHR-linked biorepositories and a coordinating center at the initiation of this study [22]. In brief, the five biorepositories included in eMERGE Phase I were: Group Health/University of Washington (GH/UW), Marshfield Clinic (MFC), Mayo Clinic (MC), Northwestern University (NU), and Vanderbilt University (VU) (Fig 1). Among the five original biorepositories, two used locally-developed EHR systems (MFC and VU), and three used commercial systems with local modifications (NU, MC, and GHC) [26]. A total of 16,029 European Americans and 2,634 African Americans were selected from eMERGE Phase I study sites and genotyped on the Illumina 660-Quad or 1M as previously described [27] and briefly described below.

In the second phase of eMERGE, the number of study sites expanded to include Children’s Hospital of Philadelphia, Boston Children’s Hospital, Cincinnati Children’s Hospital Medical Center, Geisinger Health System (GHS), and Mount Sinai School of Medicine (MSSM) [23]. In this study of adults with resistant hypertension, data from the eMERGE Phase II study sites GHS and MSSM were included (Fig 1), both of which used a commercial EHR system [23].

### Selection of resistant hypertension cases and controls

There are multiple definitions of resistant hypertension in the literature [28], including multiple subgroups and classifications of “uncontrolled” and “controlled.” International guidelines such as The National Institute for Health and Care Excellence define uncontrolled resistant hypertension as an individual with systolic or diastolic blood pressure measures >140 or >90 mm Hg, respectively, after use of three classes of anti-hypertensive medications concurrently [29]. Controlled resistant hypertension is defined by the American Heart Association Scientific Statement [7] as the concurrent use of at least four antihypertensive medication classes.

Defining any resistant hypertension group within the EHR requires a patient’s hypertensive status pre- and post- antihypertensive medication use (based on issued prescriptions) and the medication classes of the anti-hypertensives prescribed. To capture these data, two algorithms were deployed in the eMERGE Network to identify resistant hypertension cases. For the first case algorithm (“controlled case”), each case required the concurrent use of at least four antihypertensive medication classes. Medication classes considered for these definitions included angiotensin converting enzyme inhibitors or angiotensin receptor blockers, beta blockers, non-dihydropyridine calcium channel blockers, dihydropyridine calcium channel blockers, hydralazine, minoxidil, central alpha agonists, direct renin antagonists, aldosterone antagonists, alpha antagonists, and diuretics (thiazides, K-sparing, and loop diuretics) (S1 Table). Direct alpha antagonists (e.g., phentolamine and phenoxybenzamine) were excluded from the antihypertensive medication class as they are typically given to counteract pathologic adrenergic states (e.g., pheochromocytoma), which were excluded from case and control groups. For eMERGE study sites using natural language processing as part of the data extraction process for medications, the algorithm required a dose, strength, route, or frequency present with the medication name to ensure the medication mentioned represents a prescribed medication.

For the second case algorithm (“uncontrolled case”), each case required the concurrent use of at least three antihypertensive medication classes and a systolic blood pressure >140 mm Hg or diastolic blood pressure >90 mm Hg for at least one month after meeting the medication criteria. For both case definitions, patients were excluded if they had systolic heart failure (defined as an ejection fraction ≤35%) or chronic kidney disease. Chronic kidney disease was defined as an estimated glomerular filtration rate ≤30 ml/min as calculated using the Modification of Diet in Renal Disease formula [30]. Other exclusion criteria for cases and controls are given in S2 Table. Patients meeting either resistant hypertension case definitions were combined for all analyses.

Controlled hypertensives (the “controls” for this association study) were defined as patients with a measurement of systolic blood pressure >140 mm Hg or diastolic blood pressure >90 mm Hg prior to meeting the medication criteria or with ICD-9 codes for hypertension (401.*) at any time and have one medication from the medication classes described above (and never have more than one simultaneous medication class, although the medication class can change), and have all systolic and diastolic measurements <135 mg/dl and <90 mg/dl, respectively, one month after blood pressure medications were prescribed (this requires at least one blood pressure measurement). For both cases and controls, simultaneous medication class usage was defined as evidence that the patient was taking the medication concurrently based on the presence of the medications in the same medication list (e.g., problem list, clinic note, or discharge summary) or via medication refill data with accompanying evidence of overlapping prescriptions for each drug. Like the resistant hypertension cases, individuals with systolic heart failure or chronic kidney disease were excluded.

Electronic selection logic using billing codes, laboratory values, text queries, and medication records was used to identify resistant hypertension cases and controls at each site (S1 File). The initial algorithm used to identify cases and controls for this study of resistant hypertension was created and iteratively refined at VU with input from local experts as well as the eMERGE Phenotyping Workgroup and then deployed at the other study sites. At VU, physicians not associated with the algorithm development reviewed the de-identified clinical record of a fraction of identified cases and controls to assist in the refinement of the algorithm. In this iterative process, the algorithm was refined until a positive predictive value (PPV) of 92% was achieved based on reviews of 50 randomly selected clinical cases and a PPV of 84% for 50 randomly selected controls. Each round of reviews was independent and did not draw from the same set of identified cases and controls. Evaluation at the other four eMERGE I study sites confirmed PPVs of 84%-100%, including some sites that augmented the algorithmic selection with manual review. The general process of phenotype algorithm development in the eMERGE Network has been previously described [31], and the final version of workflows and algorithms are available at PheKB [32].

### Genotyping

Genotyping was performed for eMERGE I study sites using two Illumina arrays at two genotyping centers. Individuals of self-identified or administratively-assigned European-descent were genotyped on the Illumina 660W-Quad, while individuals of self-identified or administratively-assigned African-descent were genotyped on the Illumina 1M. For the majority of patients, genotyping was performed at one of two centers: the Center for Inherited Disease Research (CIDR) at Johns Hopkins University and the Center for Genotyping and Analysis at the Broad Institute as previously described [33, 34]. Existing genotype data available for eMERGE II study sites included data from the Illumina 550 (MC; n = 18), Illumina 610 (MC; n = 7), Illumina HumanOmni Express (GHS; n = 3,111), and Affymetrix 6.0 (MSSM; n = 2,775) [35].

Quality control (QC) measures were developed by the eMERGE Genomics Workgroup [33] and were implemented by the Coordinating Center. Briefly, the QC process included examination of sample quality and composition (i.e., sex inconsistency checks, sample call rates, sample relatedness, and population stratification), marker quality (i.e., marker call rate, duplicate concordance, minor allele frequency, and Hardy-Weinberg equilibrium), and of batch effects (i.e., average call rates and minor allele frequency per plate) [33, 34]. Following QC, analyses were limited to patients with call rates >98% and to SNPs with call rates >99% and minor allele frequencies >5%. Race/ethnicity was self-identified or administratively-assigned, and genetic ancestry was assessed using STRUCTURE [36] and EIGENSTRAT [37]. As reported previously, administratively-assigned race/ethnicity is highly concordant with genetic ancestry for European and African Americans [38, 39].

All genotype data were imputed by Pennsylvania State University Center for Systems Genomics as part of the eMERGE Coordinating Center as previously described [35]. Briefly, all autosomes were imputed using the 1000 Genomes cosmopolitan reference panel (n = 1,092) [40] using SHAPEIT2 [41] and IMPUTE2 [42] for phasing and imputation, respectively. Imputation was performed for each study site and genotyping assay separately and then merged for further quality control and analysis.

### Statistical methods

For the GWAS, single-SNP tests of association were performed in PLINK [43] and PLATO [44] using logistic regression and assuming an additive genetic model. All associations were adjusted for sex, decade of birth, median body mass index (BMI in kg/m^2^), genotyping platform, and genetic ancestry (via ten principal components [PCs] for multi-ethnic analyses and sex-stratified analyses, and three PCs for European-descent only analyses). Analyses were performed in the combined eMERGE I and eMERGE II datasets (3,006 cases and 876 controls). The models did not converge in the combined dataset, and inspection of the distribution of cases and controls by study site and genotyping platform revealed several strata with only case counts but no control counts. Removal of the case-only counts (231) enabled the models to converge.

The results of the GWAS tests of association were plotted using a Manhattan plot. Manhattan plots were generated by the R statistical package using code provided by the blog “Getting Genetics Done” (http://www.gettinggeneticsdone.com/2011/04/annotated-manhattan-plots-and-qq-plots.html), and regional plots were generated using LocusZoom at default settings [45]. Reported p-values were not corrected for multiple comparisons.

SNPs previously associated with blood pressure, systolic blood pressure, diastolic blood pressure, or hypertension among adults at genome-wide significance were drawn from the NHGRI European Bioinformatics Institute (NHGRI-EBI) GWAS Catalog (http://www.ebi.ac.uk/gwas/; accessed September 2016) [46, 47]. We then performed a look-up of these previously-associated blood pressure variants in the eMERGE Network resistant hypertension multi-ethnic GWAS dataset. Ad hoc power calculations were performed using CaTS [48].

## Results

### Identification of resistant hypertensive cases and controls

A total of 3,006 resistant hypertension (uncontrolled and controlled) cases and 876 controlled hypertensives were identified from the EHRs in eMERGE I and II (Table 1). Slightly more than half the cases (55%) were identified by the first case algorithm (see Methods). Cases and controls were drawn from seven study sites of eMERGE I and II, with VU (31%) and MFC (21%) contributing the largest percentage of individuals (S3 Table). For both cases and controls, nearly half were male, and the median BMI was in the overweight category (Table 1). The median birth decade was 1940 (25% and 75% quartiles were 1930 and 1940, respectively). With respect to race/ethnicity, approximately 65% of the cases were European American, and, in contrast, up 82% of controls were European American.

**Table 1:** Characteristics of resistant hypertension cases and controls. Race/ethnicity is self-reported or administratively-assigned. Here, ethnicity refers to “Hispanic” and race refers to European American, African American, or other.

	Cases (n = 3,006)	Controls (n = 876)
Male (%)	42.81	44.98
Median body mass index (kg/m^2^)	29.80	28.63
African American (%)	26.51	13.93
European American (%)	64.77	81.62
Hispanic (%)	7.05	2.51
Other (%)	1.66	1.94

### Discovery

To identify common variants associated with risk of resistant hypertension, a genome-wide association study of 2,530,150 SNPs was performed among all resistant hypertension cases (n = 2,830) and controls (n = 876) adjusted for sex, decade of birth, median BMI, genotyping platform, and ten PCs. A single association at genome-wide significance was observed between case status and *ESR1* rs9479122 at p = 6.28x10^-11^ with an odds ratio of 0.46 (S1 and S2 Figs). When restricted to European Americans only (n = 1,609 cases and n = 667 controls), *ESR1* rs9479122 remained the most significant association, p = 1.12x10^-16^ (odds ratio = 0.29; Table 2; S3–S5 Figs).

**Table 2:** SNPs associated with resistant hypertension at p<10^−6^ in the eMERGE Network in the genome-wide association study. After removal of *ESR1* rs9479122, single-SNP tests of association were performed for 2,530,149 SNPs in eMERGE I and II using logistic regression, assuming an additive genetic model, adjusted for sex, decade of birth, median body mass index, genotyping platform, and genetic ancestry (principal components 1 through 10). Results are shown for tests of association in the eMERGE I and II Network at p<10^−6^. Tests of association were repeated for European Americans only using logistic regression, assuming an additive genetic model, adjusted for sex, decade of birth, median body mass index, genotyping platform, and genetic ancestry (principal components 1 through 3). Results are also shown for European Americans for SNPs associated with resistant hypertension at p<10^−6^ in the eMERGE I and II Network. Abbreviations: basepair (bp), chromosome (chr), coded allele (CA), coded allele frequency (CAF).

SNP	Chr:BP	Gene	All eMERGE I and II (n_cases_ = 2,830; n_controls_ = 876)			European Americans only (n_cases_ = 1,719; n_controls_ = 708)		
			CAF (CA)	Odds ratio (95% CI)	P-value	CAF (CA)	Odds ratio (95% CI)	P-value
rs13144136	4:10666909	*CLNK* (intronic)	0.66 (G)	0.68 (0.58, 0.80)	1.00E-06	0.70 (G)	0.66 (0.54, 0.79)	7.42E-06
rs73007615	2:134510949	-	0.84 (C)	0.62 (0.51, 0.76)	2.89E-06	0.82 (C)	0.66 (0.53, 0.83)	2.48E-04
rs73007604	2:134506084	-	0.84 (C)	0.62 (0.51, 0.76)	3.11E-06	0.82 (C)	0.66 (0.53, 0.83)	2.48E-04
rs80028883	2:134521148	-	0.84 (T)	0.62 (0.51, 0.76)	3.43E-06	0.82 (T)	0.66 (0.53, 0.83)	2.50E-04
rs16829231	2:134531622	-	0.84 (T)	0.62 (0.51, 0.76)	3.51E-06	0.82 (T)	0.66 (0.52, 0.82)	2.37E-04
rs73005736	2:134503462	-	0.84 (G)	0.63 (0.51, 0.77)	4.07E-06	0.82 (G)	0.66 (0.53, 0.83)	3.12E-04
rs11681656	2:134495020	-	0.84 (G)	0.63 (0.52, 0.77)	5.78E-06	0.82 (G)	0.67 (0.53, 0.84)	4.41E-04
rs4533516	2:134498958	-	0.84 (C)	0.63 (0.52, 0.77)	6.27E-06	0.82 (C)	0.67 (0.53, 0.84)	4.41E-04
rs733170	2:134528895	-	0.84 (T)	0.64 (0.52, 0.78)	7.23E-06	0.82 (T)	0.67 (0.53, 0.83)	3.39E-04
rs7016717	8:78630367	-	0.89 (G)	1.77 (1.38, 2.27)	7.32E-06	0.96 (G)	1.70 (1.10, 2.63)	1.72E-02
rs12752401	1:102967150	-	0.93 (C)	0.50 (0.36, 0.68)	7.62E-06	0.92 (C)	0.49 (0.35, 0.68)	2.19E-05
rs6712538	2:134473174	-	0.83 (C)	0.64 (0.53, 0.78)	7.79E-06	0.81 (C)	0.68 (0.54, 0.84)	3.60E-04
rs6750839	2:114941169	-	0.92 (T)	0.43 (0.29, 0.64)	7.80E-06	1.00 (T)	0.97 (0.03, 28.78)	9.85E-01
rs1374404	2:134495807	-	0.84 (T)	0.64 (0.52, 0.78)	9.97E-06	0.82 (G)	0.67 (0.53, 0.84)	4.41E-04

To corroborate these potential findings, we performed a look-up for *ESR1* rs9479122 in two independent datasets, INVEST [21, 49] and SPS3 [50]. INVEST was a large hypertension outcomes trial, and SPS3 was a secondary stroke prevention trial with a blood pressure arm. Both trials conducted genetic sub-studies and resistant hypertension phenotypes were constructed in a manner similar to that constructed in eMERGE. In INVEST and SPS3, *ESR1* rs9479122 is nearly monomorphic (coded allele G frequency 0.996 and 0.99937, respectively), which is in agreement with HapMap CEU estimates. *ESR1* rs9479122 is imputed in this eMERGE dataset [35].

**Fig 2:**
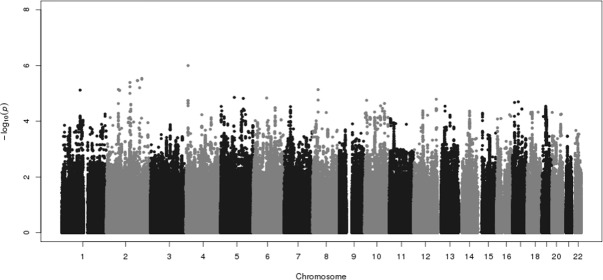
Genome-wide association analysis of individuals with resistant hypertension versus controlled hypertensives. A total of 2,830 cases of resistant hypertension and 876 controlled hypertensives from eMERGE I and II was available for analysis after quality control. After removing imputed genotypes for *ESR1* rs9479122, single-SNP tests of association were performed for 2,530,149 SNPs using logistic regression, assuming an additive genetic model, adjusted for sex, decade of birth, median body mass index, genotyping platform, and genetic ancestry (principal components 1 through 10). Results of each test of association are plotted as p-values (expressed as–log10 on the y-axis) by chromosome (x-axis).

**Fig 3:**
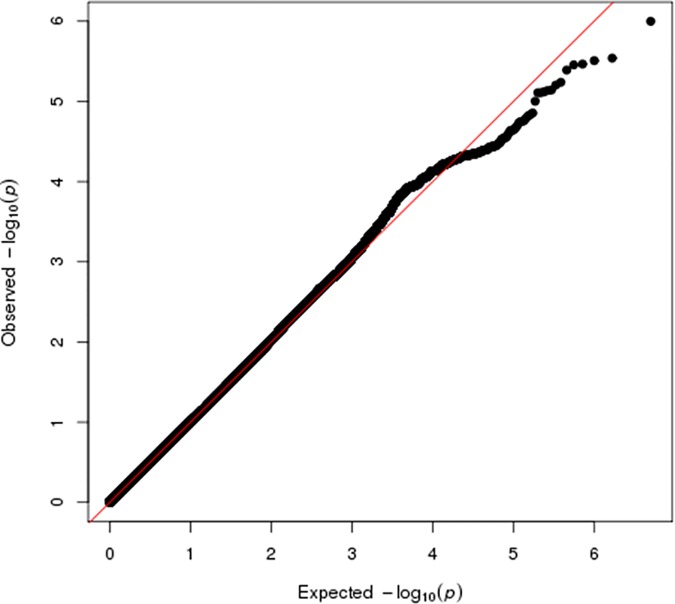
Q-Q plot of genome-wide association analysis of individuals with resistant hypertension versus controlled hypertensives. The Q-Q plots were re-generated after removal of *ESR1* rs9479122.

Given the differences in the frequency of the imputed variant in eMERGE versus reference data and the look-up datasets, we deemed *ESR1* rs9479122 a likely false positive, removed it from the dataset, and re-generated the Manhattan plots (Figs 2 and 3). No tests of association were genome-wide significant in either the overall dataset or among European Americans only (S6 and S7 Figs). In the overall dataset, the most significant finding was *CLNK* rs13144136 (odds ratio = 0.68; p = 1.00x10^-6^). *CLNK* rs13144136 was not associated with resistant hypertension in either the INVEST or SPS3 datasets (p>0.20). A total of 360 associations had nominal significance at p<10^−4^ in the present study, of which 14 were associated at p<10^−5^ (Table 2).

### Association of previously-identified hypertension and blood pressure variants with resistant hypertension

We examined whether SNPs known to influence blood pressure or hypertension also influenced resistant hypertension. The NHGRI-EBI GWAS catalog was used to select SNPs previously associated (p<5.0x10^-8^) with blood pressure, systolic blood pressure, diastolic blood pressure, or hypertension. From published GWAS papers [51–69], 118 SNPs were selected for examination in the current genome-wide studies (S4 Table). Of the 118 SNPs, 50 were available in the eMERGE I and II imputed dataset. Of the 50 tests of association performed in the present resistant hypertension study, no one association was significant (p<0.001) and in the same direction compared with the previously published literature. The most significant finding in the present study was *TBX3* rs35444, previously associated with increased diastolic blood pressure in East Asians [51], associated with resistant hypertension (eMERGE I and II combined odds ratio = 1.27; p = 1.30x10^-3^) in this multi-ethnic clinical study. With 2,830 cases and 876 controls, we were powered to replicate genetic associations for common variants (allele frequency ≥5%) with an odds ratio ≥1.5, assuming a genetic additive model and a significance threshold of 0.001. As might be expected given that the SNPs tested were from previously reported GWAS, all but one (*SLC39A8* rs13107325) of the 50 SNPs met the minor allele frequency threshold assumed in the power calculations based on the 1000 Genomes Project phase 3 genotype data from 2,500 worldwide individuals [70]. Most of the previously-reported associations tested here for associations with resistant hypertension were studies of quantitative traits (such as diastolic and systolic blood pressures) with effect sizes (β) ranging from 0.17 to 5.48 (median = 0.521). Of the few studies reporting odds ratio for associations with hypertension, only one reported an odds ratio >1.5 [71]. Although direct comparisons are difficult to make between the present study’s dichotomous outcome and previous studies’ quantitative outcomes (S4 Table), it is likely that the present study was under-powered to replicate or generalize previous findings from blood pressure GWAS.

## Discussion

We demonstrate here that DNA biobanks linked to EHRs can be used to classify resistant hypertension cases and controls and to perform large-scale genome-wide association studies. Using an algorithm based on a combination of billing codes, laboratory values, text queries, and prescription records, we were able to identify 876 controlled hypertensives and 3,006 cases of resistant hypertension from a sample of >55,000 US adults available in eMERGE Phase I and II [72]. Overall, no variants were associated with resistant hypertension at genome-wide significance in either the full dataset or the dataset limited to European Americans. Also, none of the loci previously identified for hypertension and/or blood pressure was associated with resistant hypertension in cases and controls drawn from eMERGE I and II after correction for multiple testing. It is unclear if the lack of associations observed here represent a lack of sufficient power, a lack of accurate phenotyping, or in the case of the hypertension/blood pressure-associated variants, a lack of biological relevance or connection to the phenotype of resistant hypertension.

This present work expands on earlier studies of the genetics of resistant hypertension. The handful of studies that have specifically investigated resistant hypertension have mostly focused on candidate genes: β and γ subunits of the epithelial sodium channel (*ENaC*) [17], cytochrome P450 gene (*CYP3A5*) [18], glutathione S-transferase mu type 1 gene (*GSTM1*) [16], and endothelial nitric oxide synthase gene (*NOS3*) [19, 20]. However, these studies suffered from several limitations including small sample sizes (ranging from 11 to 347 cases) and less-than-stringent significance thresholds. Of note is the large-scale candidate gene study in INVEST [21] that tested for an association between resistant hypertension (with >500 total cases) and approximately 50,000 common genetic variants targeted by the Illumina HumanCVD Genotyping BeadChip [73]. The most significant finding in INVEST (*ATP2B1* rs12817819) did not replicate in an independent dataset, but reached chip-wide significance after meta-analysis [21].

### Strengths and limitations

Major strengths of our study include the scale and longitudinal nature of the EHR. The combination of collaboration among research groups with access to biobanks and advances in high-throughput technology allowed us to genotype hundreds of thousands of genetic variants and impute to more than two million SNPs in >2,800 resistant hypertensive patients, enabling us to test for novel associations that may not have been interrogated via traditional candidate gene studies. However, compared with GWAS of common diseases, studies requiring exposure to medications or other treatments face additional challenges [74]. While GWAS of common diseases such as type 2 diabetes [75, 76] or lipid levels [77, 78] are able to accrue tens to hundreds of thousands of individuals for study, obtaining adequate numbers for some clinical traits is challenging as observational cohorts often do not have sufficient data related to drug exposure or treatments, and existing sets of genotyped clinical records are still relatively small. Indeed, even with our fairly large sample size, we were still underpowered to detect small effects at genome-wide significance. Specifically, for common SNPs (minor allele frequency = 0.20), we had 80% power to detect an odds ratio of 1.41.

Statistical power has been a major obstacle for studies designed to identify genetic variants associated with blood pressure in general. Indeed, even contemporary studies with access to genome-wide and imputed data of millions of common variants estimate that one-third to one-half the variability observed for systolic and diastolic blood pressure levels can be attributed to the additive effects of many genetic variants; to date, only ~2% of blood pressure variation can be explained by known blood pressure-associated variants [79], each with very small contributions, thus requiring large sample sizes to detect the associations via GWAS [79]. Given the genetic and environmental complexity of blood pressure, it is not surprising that the present study was unable to identify novel variants associated with resistant hypertension.

The upcoming NIH Precision Medicine Initiative Cohort Program (“All of Us”), which seeks to accrue >1 million individuals with longitudinal EHR data, may facilitate future studies on drug effects on a scale not possible today [80, 81]. Also, longitudinal studies such as those proposed by the NIH Precision Medicine Initiative Cohort Program increase the chances that secondary causes of resistant hypertension will be identified and eliminated to reduce phenotypic heterogeneity. However, it is worth noting that while potentially large, the Precision Medicine Initiative Cohort Program may suffer from similar limitations experienced by the individual study sites of the eMERGE Network. That is, the eMERGE Network is large in overall sample size, but the requirements of repeated blood pressure measurements and prescription information over the course of time severely limited the case and control counts available for the association study. This dramatic loss of sample size and resulting loss of power is a reflection of the fragmented health care system in the United States. Studies requiring extensive longitudinal data may be better powered in health care settings where patients regularly receive the vast majority of his or her care within a single setting such as an integrated managed care consortium (Kaiser Permanente [82, 83]) or the Veterans Health Administration [84].

Phenotypic heterogeneity in general is a major challenge faced by most studies [74]. Our study is no exception. While our algorithm and subsequent review removed potential sources of case misclassification (such as acute myocardial infarction patients on more than one class of medication), it is important to note that elevated blood pressure readings may result from measurement error, other concurrent medical conditions (e.g., pain or anxiety), poor patient compliance, or “white-coat hypertension.” Patients who lack blood pressure control despite appropriate treatment due to the aforementioned reasons may present with resistant hypertension but are actually “pseudoresistant” [9]. Potential lifestyle causes of resistant hypertension, such as excessive dietary salt intake and heavy alcohol use, are also not measured and recorded in the EHR. While large-scale biobanks linked to EHRs offer important advantages in research settings, misclassification bias must be recognized and minimized by carefully characterizing the phenotype and exploiting the longitudinal nature of the EHR [85].

Of special note is the issue of poor compliance or non-compliance. Most studies involving medications or self-administered treatments have a problem with non-compliance. For hypertension, clinical and epidemiologic surveys continuously suggest that a high proportion of patients do not achieve target blood pressure levels after treatment [86, 87]. This observed resistance to treatment can be reflective of true resistance, the outcome of interest in the present study, or apparent resistance which often times is reflective of poor adherence to treatment. Previous retrospective studies have noted that approximately 40% of newly diagnosed hypertensive patients discontinue treatment within a year [7, 88, 89]. Multiple factors have been associated with poor adherence to hypertensive treatment or treatment in general including non-patient (such as drug class [90]) and patient factors (such as sex, race/ethnicity, and socio-economic status [91]), and some data suggest that women prescribed anti-hypertensives are more compliant than men [92]. Nearly half the sample in this present study was male. Additional information such as prescription claims data could help clarify this issue of non-compliance.

Finally, a major challenge in designing optimal studies to identify and characterize the genetic architecture of resistant hypertension may be related to the as-of-yet unknown etiology of the phenotype(s) [10]. For example, more traditional pharmacogenomics studies of blood pressure and response to anti-hypertensive treatments concentrate on a single class of drug (such as hydrochlorothiazide monotherapy [93]) representing a specific pathway or mechanism of action. It could be argued that resistant hypertension represents the dichotomized version of the extremes of the drug-response distribution as the majority of resistant hypertension cases studied here required four medications from different classes. It is unlikely that a single genetic variant affects all pathways represented by these medication classes but rather a complex combination or interaction of variants that phenotypically result in resistant hypertension. This genetic heterogeneity in combination with the aforementioned phenotypic heterogeneity and phenotyping requirements makes the genetic study of resistant hypertension especially challenging.

In conclusion, we describe an approach to identify resistant hypertension cases and controls from seven different institutions’ EHRs. To our knowledge, this is the only GWAS of resistant hypertension in any population. These results highlight the utility of EHR-linked genomic data to further refine our understanding of disease and its subtypes, with the goal of uncovering new therapeutic targets and providing better targeting of current therapies.

## Supporting information

S1 Fig Likely false-positive association in genome-wide association analysis of individuals with resistant hypertension versus controlled hypertensives. A total of 876 cases of resistant hypertension and 2,830 controlled hypertensives from the eMERGE I and II Network were available for analysis. Single-SNP tests of association were performed for 2,530,150 SNPs using logistic regression, assuming an additive genetic model, adjusted for sex, decade of birth, median body mass index, genotyping platform, and genetic ancestry (principal components 1 through 10). Results of each test of association are plotted as p-values (expressed as–log10 on the y-axis) by chromosome (x-axis). The most significant finding was *ESR1* rs9479122 on chromosome 6, a likely false positive due to poor genotyping for this variant.
(DOCX)

S2 Fig Q-Q plot of genome-wide association analysis of individuals with resistant hypertension versus controlled hypertensives. The most significant finding (*ESR1* rs9479122) is likely a false-positive due to poor genotyping prior to imputation and was removed.
(DOCX)

S3 Fig Genome-wide association study of European Americans with resistant hypertension versus controlled hypertensives. A total of 2,530,150 SNPs were tested for an association with resistant hypertension (1,719 cases and 708 controls) among Europeans from the eMERGE I and II Network. Tests of association were performed using logistic regression assuming an additive genetic model and adjusting for sex, decade of birth, genotyping platform, median body mass index, and principal components (1–3). Each test of association was plotted where the x-axis is the chromosomal location and the y-axis is–log_10_ of the p-value. The most significant finding was *ESR1* rs9479122 on chromosome 6, a likely false positive due to poor genotyping for this variant.
(DOCX)

S4 Fig Q-Q plot of genome-wide association study of European Americans with resistant hypertension versus controlled hypertensives. A total of 2,530,150 SNPs were tested for an association with resistant hypertension (1,719 cases and 708 controls) among Europeans from the eMERGE I and II Network. Tests of association were performed using logistic regression assuming an additive genetic model and adjusting for sex, decade of birth, genotyping platform, median body mass index, and principal components (1–3). The most significant finding (*ESR1* rs9479122) is likely a false-positive due to poor genotyping prior to imputation and was removed.
(DOCX)

S5 Fig LocusZoon plot of genome-wide significant result in *ESR1* in European Americans with resistant hypertension versus controlled hypertensives. A genome-wide association study was performed for 1,719 European American cases of resistant hypertension and 708 controls from the eMERGE Network adjusted for sex, decade of birth, median body mass index, genotyping platform, and genetic ancestry (principal components 1–3). The left x-axis is the–log_10_(p-value) of the tests of association and the right x-axis is the recombination rate (cM/Mb). The most significant result in European Americans (rs9479122) is plotted as the index variant color-coded as a purple diamond. Surrounding SNPs are circles color-coded by strength of linkage disequilibrium (LD calculated as r^2^), where red is complete or very strong LD and blue is weak LD or independent variants. LD was calculated using HapMap CEU data (release 22) in the default version of LocusZoom. Gene names and position on chromosome 6 (Mb) are given on the y-axis. The most significant finding (*ESR1* rs9479122) is likely a false-positive due to poor genotyping prior to imputation and was removed.
(DOCX)

S6 Fig Genome-wide association study of European Americans with resistant hypertension versus controlled hypertensives. A total of 2,530,150 SNPs were tested for an association with resistant hypertension (1,719 cases and 708 controls) among Europeans from the eMERGE I and II network. After removal of *ESR1* rs9479122, tests of association were performed using logistic regression assuming an additive genetic model and adjusting for sex, decade of birth, genotyping platform, median body mass index, and principal components (1–3). Each test of association was plotted on a Manhattan plot where the x-axis is the chromosomal location and the y-axis is–log_10_ of the p-value.
(DOCX)

S7 Fig Q-Q plot of genome-wide association study of European Americans with resistant hypertension versus controlled hypertensives. A total of 2,530,150 SNPs were tested for an association with resistant hypertension (1,719 cases and 708 controls) among Europeans from the eMERGE I and II network. After removal of *ESR1* rs9479122, tests of association were performed using logistic regression assuming an additive genetic model and adjusting for sex, decade of birth, genotyping platform, median body mass index, and principal components (1–3).
(DOCX)

S1 File electronic MEdical Records & GEnomics (eMERGE) Network Resistant Hypertension electronic health record (EHR) study inclusion and exclusion criteria. (DOCX)

S1 Table Medication classes considered in defining resistant hypertension status in the electronic MEdical Records & GEnomics (eMERGE) Network. (DOCX)

S2 Table Exclusions from case and control definitions of resistant hypertension in the eMERGE Network. Individuals were excluded from case or control status based on ICD-9-CM codes and situations as described. In addition to exclusions based on codes, individuals were excluded from case status if there was evidence of chronic kidney disease within six months after meeting the definition for “controlled” resistant hypertension (four medication classes concurrently) or heart failure within one year before or after meeting the definition for “controlled” resistant hypertension. Chronic kidney disease was defined by an estimated glomerular filtration rate (eGFR) ≤30 ml/min, as calculated by the Modification of Diet in Renal Disease formula. Heart failure was defined as an ejection fraction (EF) or left ventricular ejection fraction (LVEF) ≤35%. Individuals with evidence of heart failure were also excluded from control status.
(DOCX)

S3 Table Resistant hypertension cases and controls for discovery, by eMERGE study site and population. Counts within parentheses represent number of additional samples identified as cases or controls but not included in the final analyses due to model convergence issues.
(DOCX)

S4 Table Variants previously identified in GWAS of blood pressure or hypertension in current GWAS of resistant hypertension. The “Published GWAS” columns represent SNPs previously associated with blood pressure, systolic blood pressure, diastolic blood pressure, or hypertension among adults at genome-wide significance drawn from the NHGRI European Bioinformatics Institute (NHGRI-EBI) GWAS Catalog (http://www.ebi.ac.uk/gwas/; accessed September 2016). Published GWAS data are compared with eMERGE I and II association results for resistant hypertension among all racial/ethnic adults if the SNP is present in the dataset. Abbreviations: beta (β), p (p-value), odds ratio (OR), lower 95% confidence interval (L95), and upper 95% confidence interval (U95). Data from eMERGE I and eMERGE II are denoted by the subscripts.
(DOCX)

## References

[1] Guo F, He D, Zhang W, Walton RG. Trends in Prevalence, Awareness, Management, and Control of Hypertension Among United States Adults, 1999 to 2010. Journal of the American College of Cardiology. 2012;60(7):599–606. 10.1016/j.jacc.2012.04.026 22796254

[2] Fields LE, Burt VL, Cutler JA, Hughes J, Roccella EJ, Sorlie P. The Burden of Adult Hypertension in the United States 1999 to 2000: A Rising Tide. Hypertension. 2004;44(4):398–404. 10.1161/01.HYP.0000142248.54761.56 15326093

[3] Lenfant C. Reflections on hypertension control rates: A message from the director of the national heart, lung, and blood institute. Archives of Internal Medicine. 2002;162(2):131–2. 11802745 10.1001/archinte.162.2.131

[4] Chobanian AV, Bakris GL, Black HR, Cushman WC, Green LA, Izzo JL, et al Seventh Report of the Joint National Committee on Prevention, Detection, Evaluation, and Treatment of High Blood Pressure. Hypertension. 2003;42(6):1206–52. 10.1161/01.HYP.0000107251.49515.c2 14656957

[5] Go AS, Mozaffarian D, Roger VL, Benjamin EJ, Berry JD, Blaha MJ, et al Heart Disease and Stroke Statistics—2014 Update. A Report From the American Heart Association. 2014;129(3):e28–e292. 10.1161/01.cir.0000441139.02102.80 PMC5408159 24352519

[6] Bakris G, Sarafidis P, Agarwal R, Ruilope L. Review of blood pressure control rates and outcomes. Journal of the American Society of Hypertension. 2014;8(2):127–41. 10.1016/j.jash.2013.07.009 24309125

[7] Calhoun DA, Jones D, Textor S, Goff DC, Murphy TP, Toto RD, et al Resistant Hypertension: Diagnosis, Evaluation, and Treatment: A Scientific Statement From the American Heart Association Professional Education Committee of the Council for High Blood Pressure Research. Circulation. 2008;117(25):e510–e26. 10.1161/CIRCULATIONAHA.108.189141 18574054

[8] Sarafidis PA, Georgianos P, Bakris GL. Resistant hypertension—its identification and epidemiology. Nat Rev Nephrol. 2013;9(1):51–8. 10.1038/nrneph.2012.260 23165303

[9] Sarafidis PA, Bakris GL. Resistant Hypertension: An Overview of Evaluation and Treatment. Journal of the American College of Cardiology. 2008;52(22):1749–57. 10.1016/j.jacc.2008.08.036 19022154

[10] El Rouby N, Cooper-DeHoff R. Genetics of Resistant Hypertension: a Novel Pharmacogenomics Phenotype. Curr Hypertens Rep. 2015;17(9):1–11. 10.1007/s11906-015-0583-8 PMC4717470 26198781

[11] Franceschini N, Reiner AP, Heiss G. Recent Findings in the Genetics of Blood Pressure and Hypertension Traits. American Journal of Hypertension. 2011;24(4):392–400. 10.1038/ajh.2010.218 20948529 PMC3110743

[12] Binder A. A review of the genetics of essential hypertension. Curr Opin Cardiol. 2007;22(3):176–84. 10.1097/HCO.0b013e3280d357f9 17413273

[13] Johnson JA. Advancing management of hypertension through pharmacogenomics. Annals of Medicine. 2012;44(sup1):S17–S22. 22713143 10.3109/07853890.2011.653399 PMC3686275

[14] Lupton SJ, Chiu CL, Lind JM. A hypertension gene: are we there yet? Twin Res Hum Genet. 2011;14(4):295–304. 10.1375/twin.14.4.295 21787111

[15] Gong Y, McDonough CW, Beitelshees AL, El Rouby N, Hiltunen TP, O'Connell JR, et al PTPRD gene associated with blood pressure response to atenolol and resistant hypertension. J Hyertens. 2015;33(11):2278–85. 10.1097/HJH.0000000000000714 PMC4788379 26425837

[16] Cruz-Gonzalez I, Corral E, Sanchez-Ledesma M, Sanchez-Rodriguez A, Martin-Luengo C, Gonzalez-Sarmiento R. An association between resistant hypertension and the null GSTM1 genotype. J Hum Hypertens. 2009;23(8):556–8. 10.1038/jhh.2009.19 19279659

[17] Hannila-Handelberg T, Kontula K, Tikkanen I, Tikkanen T, Fyhrquist F, Helin K, et al Common variants of the beta and gamma subunits of the epithelial sodium channel and their relation to plasma renin and aldosterone levels in essential hypertension. BMC Medical Genetics. 2005;6(1):1–13. 15661075 10.1186/1471-2350-6-4 PMC547905

[18] Ho H, Pinto A, Hall SD, Flockhart DA, Li L, Skaar TC, et al Association Between the CYP3A5 Genotype and Blood Pressure. Hypertension. 2005;45(2):294–8. 10.1161/01.HYP.0000151361.31736.96 15596575

[19] Cruz-González I, Corral E, Sánchez-Ledesma M, Sánchez-Rodríguez A, Martín-Luengo C, González-Sarmiento R. Association between -T786C NOS3 polymorphism and resistant hypertension: a prospective cohort study. BMC Cardiovascular Disorders. 2009;9(1):1–6. 19650939 10.1186/1471-2261-9-35 PMC2734743

[20] Jáchymová M, Horký K, Bultas J, Kožich V, Jindra A, Peleška J, et al Association of the Glu298Asp Polymorphism in the Endothelial Nitric Oxide Synthase Gene with Essential Hypertension Resistant to Conventional Therapy. Biochemical and Biophysical Research Communications. 2001;284(2):426–30. 10.1006/bbrc.2001.5007 11394896

[21] Fontana V, McDonough CW, Gong Y, El Rouby NM, Sa AC, Taylor KD, et al Large-scale gene-centric analysis identifies polymorphisms for resistant hypertension. J Am Heart Assoc. 2014;3(6):e001398 10.1161/JAHA.114.001398 25385345 PMC4338734

[22] McCarty C, Chisholm R, Chute C, Kullo I, Jarvik G, Larson E, et al The eMERGE Network: A consortium of biorepositories linked to electronic medical records data for conducting genomic studies. BMC Medical Genomics. 2011;4(1):13. 21269473 10.1186/1755-8794-4-13 PMC3038887

[23] Gottesman O, Kuivaniemi H, Tromp G, Faucett WA, Li R, Manolio TA, et al The Electronic Medical Records and Genomics (eMERGE) Network: past, present, and future. Genet Med. 2013;15(10):761–71. 10.1038/gim.2013.72 23743551 PMC3795928

[24] Roden DM, Pulley JM, Basford MA, Bernard GR, Clayton EW, Balser JR, et al Development of a Large-Scale De-Identified DNA Biobank to Enable Personalized Medicine. Clin Pharmacol Ther. 2008;84(3):362–9. 10.1038/clpt.2008.89 18500243 PMC3763939

[25] Pulley J, Clayton E, Bernard GR, Roden DM, Masys DR. Principles of Human Subjects Protections Applied in an Opt-Out, De-identified Biobank. Clinical and Translational Science. 2010;3(1):42–8. 10.1111/j.1752-8062.2010.00175.x 20443953 PMC3075971

[26] Kho AN, Pacheco JA, Peissig PL, Rasmussen L, Newton KM, Weston N, et al Electronic Medical Records for Genetic Research: Results of the eMERGE Consortium. Science Translational Medicine. 2011;3(79):79re1–re1. 10.1126/scitranslmed.3001807 21508311 PMC3690272

[27] Denny JC, Crawford DC, Ritchie MD, Bielinski SJ, Basford MA, Bradford Y, et al Variants near FOXE1 are associated with hypothyroidism and other thyroid conditions: using electronic medical records for genome- and phenome-wide studies. Am J Hum Genet. 2011;89(4):529–42. 10.1016/j.ajhg.2011.09.008 21981779 PMC3188836

[28] White WB, Turner JR, Sica DA, Bisognano JD, Calhoun DA, Townsend RR, et al. Detection, evaluation, and treatment of severe and resistant hypertension: Proceedings from an American Society of Hypertension Interactive Forum held in Bethesda, MD, USA, October 10th 2013. Journal of the American Society of Hypertension. 2014;8(10):743–57. 10.1016/j.jash.2014.06.005 25418497

[29] Myat A, Redwood SR, Qureshi AC, Spertus JA, Williams B. Resistant hypertension. BMJ. 2012;345. 10.1136/bmj.e7473 23169802

[30] Levey AS, Bosch JP, Lewis JB, Greene T, Rogers N, Roth D. A More Accurate Method To Estimate Glomerular Filtration Rate from Serum Creatinine: A New Prediction Equation. Annals of Internal Medicine. 1999;130(6):461–70. 10075613 10.7326/0003-4819-130-6-199903160-00002

[31] Newton KM, Peissig PL, Kho AN, Bielinski SJ, Berg RL, Choudhary V, et al Validation of electronic medical record-based phenotyping algorithms: results and lessons learned from the eMERGE network. Journal of the American Medical Informatics Association. 2013;20(e1):e147–e54. 10.1136/amiajnl-2012-000896 23531748 PMC3715338

[32] Kirby JC, Speltz P, Rasmussen LV, Basford M, Gottesman O, Peissig PL, et al PheKB: a catalog and workflow for creating electronic phenotype algorithms for transportability. Journal of the American Medical Informatics Association. 2016. 10.1093/jamia/ocv202 PMC5070514 27026615

[33] Turner S, Armstrong LL, Bradford Y, Carlson CS, Crawford DC, Crenshaw AT, et al Quality Control Procedures for Genome-Wide Association Studies. Curr Protoc Hum Genet. 2011;68(1.18):1–19. PMC3066182. 10.1002/0471142905.hg0119s68 PMC3066182 21234875

[34] Zuvich RL, Armstrong LL, Bielinski SJ, Bradford Y, Carlson CS, Crawford DC, et al Pitfalls of merging GWAS data: lessons learned in the eMERGE network and quality control procedures to maintain high data quality. Genetic Epidemiology. 2011;35(8):887–98. 10.1002/gepi.20639 22125226 PMC3592376

[35] Verma SS, de Andrade M, Tromp G, Kuivaniemi H, Pugh E, Namjou-Khales B, et al Imputation and quality control steps for combining multiple genome-wide datasets. Frontiers in Genetics. 2014;5:370 10.3389/fgene.2014.00370 25566314 PMC4263197

[36] Pritchard JK, Stephens M, Donnelly P. Inference of Population Structure Using Multilocus Genotype Data. Genetics. 2000;155(2):945–59. 10835412 10.1093/genetics/155.2.945 PMC1461096

[37] Price AL, Patterson NJ, Plenge RM, Weinblatt ME, Shadick NA, Reich D. Principal components analysis corrects for stratification in genome-wide association studies. Nat Genet. 2006;38(8):904–9. 10.1038/ng1847 16862161

[38] Dumitrescu L, Ritchie MD, Brown-Gentry K, Pulley JM, Basford M, Denny JC, et al Assessing the accuracy of observer-reported ancestry in a biorepository linked to electronic medical records. Genet Med. 2010;12(10):648–50. PMC2952033. 10.1097/GIM.0b013e3181efe2df 20733501 PMC2952033

[39] Hall JB, Dumitrescu L, Dilks HH, Crawford DC, Bush WS. Accuracy of Administratively-Assigned Ancestry for Diverse Populations in an Electronic Medical Record-Linked Biobank. PLoS ONE. 2014;9(6):e99161 PMC4045967. 10.1371/journal.pone.0099161 24896101 PMC4045967

[40] An integrated map of genetic variation from 1,092 human genomes. Nature. 2012;491(7422):56–65. 10.1038/nature11632 23128226 PMC3498066

[41] Delaneau O, Zagury JF, Marchini J. Improved whole-chromosome phasing for disease and population genetic studies. Nat Meth. 2013;10(1):5–6. 10.1038/nmeth.2307 23269371

[42] Howie B, Fuchsberger C, Stephens M, Marchini J, Abecasis GR. Fast and accurate genotype imputation in genome-wide association studies through pre-phasing. Nat Genet. 2012;44(8):955–9. 10.1038/ng.2354 22820512 PMC3696580

[43] Purcell S, Neale B, Todd-Brown K, Thomas L, Ferreira MA, Bender D, et al PLINK: a tool set for whole-genome association and population-based linkage analysis. Am J Hum Genet. 2007;81(3):559–75. 10.1086/519795 17701901 PMC1950838

[44] Grady BJ, Torstenson E, Dudek SM, Giles J, Sexton D, Ritchie MD. Finding unique filter sets in PLATO: a precursor to efficient interaction analysis in GWAS data. Pac Symp Biocomput. 2010:315–26. 19908384 PMC2903053

[45] Pruim RJ, Welch RP, Sanna S, Teslovich TM, Chines PS, Gliedt TP, et al LocusZoom: regional visualization of genome-wide association scan results. Bioinformatics. 2010;26(18):2336–7. 10.1093/bioinformatics/btq419 20634204 PMC2935401

[46] Hindorff LA, Sethupathy P, Junkins HA, Ramos EM, Mehta JP, Collins FS, et al Potential etiologic and functional implications of genome-wide association loci for human diseases and traits. Proceedings of the National Academy of Sciences. 2009;106(23):9362–7. 10.1073/pnas.0903103106 PMC2687147 19474294

[47] Welter D, MacArthur J, Morales J, Burdett T, Hall P, Junkins H, et al The NHGRI GWAS Catalog, a curated resource of SNP-trait associations. Nucleic Acids Research. 2014;42(D1):D1001–D6. 24316577 10.1093/nar/gkt1229 PMC3965119

[48] Skol AD, Scott LJ, Abecasis GR, Boehnke M. Joint analysis is more efficient than replication-based analysis for two-stage genome-wide association studies. Nat Genet. 2006;38(2):209–13. 10.1038/ng1706 16415888

[49] Pepine CJ, Handberg EM, Cooper-DeHoff RM, et al A calcium antagonist vs a non–calcium antagonist hypertension treatment strategy for patients with coronary artery disease: The international verapamil-trandolapril study (invest): a randomized controlled trial. JAMA. 2003;290(21):2805–16. 10.1001/jama.290.21.2805 14657064

[50] The SPSSG. Blood-pressure targets in patients with recent lacunar stroke: the SPS3 randomised trial. The Lancet. 382(9891):507–15. 10.1016/S0140-6736(13)60852-1 PMC3979302 23726159

[51] Kato N, Takeuchi F, Tabara Y, Kelly TN, Go MJ, Sim X, et al Meta-analysis of genome-wide association studies identifies common variants associated with blood pressure variation in east Asians. Nat Genet. 2011;43(6):531–8. 10.1038/ng.834 21572416 PMC3158568

[52] Levy D, Ehret GB, Rice K, Verwoert GC, Launer LJ, Dehghan A, et al Genome-wide association study of blood pressure and hypertension. Nat Genet. 2009;41(6):677–87. 10.1038/ng.384 19430479 PMC2998712

[53] Franceschini N, Fox E, Zhang Z, Edwards T-á, Nalls M-á, Sung Y-á, et al Genome-wide Association Analysis of Blood-Pressure Traits in African-Ancestry Individuals Reveals Common Associated Genes in African and Non-African Populations. The American Journal of Human Genetics. 2013;93(3):545–54. PMC3769920. 10.1016/j.ajhg.2013.07.010 23972371 PMC3769920

[54] Lu X, Wang L, Lin X, Huang J, Charles Gu C, He M, et al Genome-wide association study in Chinese identifies novel loci for blood pressure and hypertension. Human Molecular Genetics. 2015;24(3):865–74. 10.1093/hmg/ddu478 25249183 PMC4303798

[55] Sung YJ, de Las Fuentes L, Schwander KL, Simino J, Rao DC. Gene-smoking interactions identify several novel blood pressure loci in the Framingham Heart Study. Am J Hypertens. 2015;28(3):343–54. PMC4402348. 10.1093/ajh/hpu149 25189868 PMC4402348

[56] Simino J, Shi G, Bis Joshua C, Chasman Daniel I, Ehret Georg B, Gu X, et al Gene-Age Interactions in Blood Pressure Regulation: A Large-Scale Investigation with the CHARGE, Global BPgen, and ICBP Consortia. The American Journal of Human Genetics. 2014;95(1):24–38. PMC4085636. 10.1016/j.ajhg.2014.05.010 24954895 PMC4085636

[57] Kim YK, Kim Y, Hwang MY, Shimokawa K, Won S, Kato N, et al Identification of a genetic variant at 2q12.1 associated with blood pressure in East-Asians by genome-wide scan including gene-environment interactions. BMC Medical Genetics. 2014;15(1):1–8. PMC4059884. 24903457 10.1186/1471-2350-15-65 PMC4059884

[58] He J, Kelly TN, Zhao Q, Li H, Huang J, Wang L, et al Genome-Wide Association Study Identifies 8 Novel Loci Associated With Blood Pressure Responses to Interventions in Han Chinese. Circulation: Cardiovascular Genetics. 2013;6(6):598–607. PMC3952335. 10.1161/CIRCGENETICS.113.000307 24165912 PMC3952335

[59] Hong K-W, Kim SS, Kim Y. Genome-Wide Association Study of Orthostatic Hypotension and Supine-Standing Blood Pressure Changes in Two Korean Populations. Genomics Inform. 2013;11(3):129–34. PMC3794085. 10.5808/GI.2013.11.3.129 24124408 PMC3794085

[60] Kelly TN, Takeuchi F, Tabara Y, Edwards TL, Kim YJ, Chen P, et al Genome-Wide Association Study Meta-Analysis Reveals Transethnic Replication of Mean Arterial and Pulse Pressure Loci. Hypertension. 2013;62(5):853–9. PMC3972802. 10.1161/HYPERTENSIONAHA.113.01148 24001895 PMC3972802

[61] Melka MG, Bernard M, Mahboubi A, Abrahamowicz M, Paterson AD, Syme C, et al Genome-wide scan for loci of adolescent obseity and their relationship with blood pressure. J Clin Endocrinol Metab. 2012;97(1):E145–50. 10.1210/jc.2011-1801 22013104

[62] Ehret GB, Munroe PB, Rice KM, Bochud M, Johnson AD, Chasman DI, et al Genetic variants in novel pathways influence blood pressure and cardiovascular disease risk. Nature. 2011;478. 10.1038/nature10405 PMC3340926 21909115

[63] Wain LV, Verwoert GC, O'Reilly PF, Shi G, Johnson T, Johnson AD, et al Genome-wide association study identifies six new loci influencing pulse pressure and mean arterial pressure. Nat Genet. 2011;43(10):1005–11. PMC3445021. 10.1038/ng.922 21909110 PMC3445021

[64] Blech I, Katzenellenbogen M, Katzenellenbogen A, Wainstein J, Rubinstein A, Harman-Boehm I, et al Predicting Diabetic Nephropathy Using a Multifactorial Genetic Model. PLoS ONE. 2011;6(4):e18743 10.1371/journal.pone.0018743 21533139 PMC3077408

[65] Slavin TP, Feng T, Schnell A, Zhu X, Elston RC. Two-marker association tests yield new disease associations for coronary artery disease and hypertension. Human Genetics. 2011;130(6):725–33. PMC3576836. 10.1007/s00439-011-1009-6 21626137 PMC3576836

[66] Kraja AT, Vaidya D, Pankow JS, Goodarzi MO, Assimes TL, Kullo IJ, et al A Bivariate Genome-Wide Approach to Metabolic Syndrome. STAMPEED Consortium. 2011;60(4):1329–39. PMC3064107. 10.2337/db10-1011 PMC3064107 21386085

[67] Lettre G, Palmer CD, Young T, Ejebe KG, Allayee H, Benjamin EJ, et al Genome-Wide Association Study of Coronary Heart Disease and Its Risk Factors in 8,090 African Americans: The NHLBI CARe Project. PLoS Genet. 2011;7(2):e1001300 10.1371/journal.pgen.1001300 21347282 PMC3037413

[68] Padmanabhan S, Melander O, Johnson T, Di Blasio AM, Lee WK, Gentilini D, et al Genome-Wide Association Study of Blood Pressure Extremes Identifies Variant near UMOD Associated with Hypertension. PLoS Genet. 2010;6(10):e1001177 PMC2965757. 10.1371/journal.pgen.1001177 21082022 PMC2965757

[69] Newton-Cheh C, Johnson T, Gateva V, Tobin MD, Bochud M, Coin L, et al Genome-wide association study identifies eight loci associated with blood pressure. Nat Genet. 2009;41. PMC2891673. 10.1038/ng.361 PMC2891673 19430483

[70] Consortium GP. A global reference for human genetic variation. Nature. 2015;526(7571):68–74. 10.1038/nature15393 26432245 PMC4750478

[71] Kim SJ, Lee SK, Kim SH, Yun C-H, Kim JH, Thomas RJ, et al Genetic Association of Short Sleep Duration With Hypertension Incidence—A 6-Year Follow-up in the Korean Genome and Epidemiology Study. Circulation Journal. 2012;76(4):907–13. 22322875 10.1253/circj.cj-11-0713

[72] Crawford DC, Crosslin DR, Tromp G, Kullo IJ, Kuivaniemi H, Hayes MG, et al eMERGEing progress in genomics—the first seven years. Frontiers in Genetics. 2014;5:184 PMC4060012. 10.3389/fgene.2014.00184 24987407 PMC4060012

[73] Keating BJ, Tischfield S, Murray SS, Bhangale T, Price TS, Glessner JT, et al Concept, Design and Implementation of a Cardiovascular Gene-Centric 50 K SNP Array for Large-Scale Genomic Association Studies. PLoS ONE. 2008;3(10):e3583 10.1371/journal.pone.0003583 18974833 PMC2571995

[74] Daly AK. Using Genome-Wide Association Studies to Identify Genes Important in Serious Adverse Drug Reactions. Annual Review of Pharmacology and Toxicology. 2012;52(1):21–35. 10.1146/annurev-pharmtox-010611-134743 21819236

[75] Voight BF, Scott LJ, Steinthorsdottir V, Morris AP, Dina C, Welch RP, et al Twelve type 2 diabetes susceptibility loci identified through large-scale association analysis. Nat Genet. 2010;42(7):579–89. 10.1038/ng.609 20581827 PMC3080658

[76] Consortium DGRAM-aD, Type AGEN, South AT, Mexican AT, (DGEbN-gsim-ES, Mahajan A, et al Genome-wide trans-ancestry meta-analysis provides insight into the genetic architecture of type 2 diabetes susceptibility. Nat Genet. 2014;46(3):234–44. 10.1038/ng.2897 24509480 PMC3969612

[77] Teslovich TM, Musunuru K, Smith AV, Edmondson AC, Stylianou IM, Koseki M, et al Biological, clinical and population relevance of 95 loci for blood lipids. Nature. 2010;466(7307):707–13. 10.1038/nature09270 20686565 PMC3039276

[78] Global Lipids Genetics C. Discovery and refinement of loci associated with lipid levels. Nat Genet. 2013;45(11):1274–83. http://www.nature.com/ng/journal/v45/n11/abs/ng.2797.html#supplementary-information. 10.1038/ng.2797 24097068 PMC3838666

[79] Salfati E, Morrison AC, Boerwinkle E, Chakravarti A. Direct Estimates of the Genomic Contributions to Blood Pressure Heritability within a Population-Based Cohort (ARIC). PLoS ONE. 2015;10(7):e0133031 10.1371/journal.pone.0133031 26162070 PMC4498745

[80] Collins FS, Varmus H. A New Initiative on Precision Medicine. New England Journal of Medicine. 2015;372(9):793–5. 10.1056/NEJMp1500523 25635347 PMC5101938

[81] Group PMIPW. The Precision Medicine Initiative Cohort Program—Building a Research Foundation for 21st Century Medicine. 2015 9/17/2015. Report No.

[82] Banda Y, Kvale MN, Hoffmann TJ, Hesselson SE, Ranatunga D, Tang H, et al Characterizing Race/Ethnicity and Genetic Ancestry for 100,000 Subjects in the Genetic Epidemiology Research on Adult Health and Aging (GERA) Cohort. Genetics. 2015;200(4):1285–95. 10.1534/genetics.115.178616 26092716 PMC4574246

[83] Kvale MN, Hesselson S, Hoffmann TJ, Cao Y, Chan D, Connell S, et al Genotyping Informatics and Quality Control for 100,000 Subjects in the Genetic Epidemiology Research on Adult Health and Aging (GERA) Cohort. Genetics. 2015;200(4):1051–60. 10.1534/genetics.115.178905 26092718 PMC4574249

[84] Gaziano JM, Concato J, Brophy M, Fiore L, Pyarajan S, Breeling J, et al Million Veteran Program: A mega-biobank to study genetic influences on health and disease. Journal of Clinical Epidemiology. 2016. 10.1016/j.jclinepi.2015.09.016 26441289

[85] Roden DM, Xu H, Denny JC, Wilke RA. Electronic Medical Records as a Tool in Clinical Pharmacology: Opportunities and Challenges. Clinical Pharmacology & Therapeutics. 2012;91(6):1083–6. 22534870 10.1038/clpt.2012.42 PMC3819803

[86] Ong KL, Cheung BMY, Man YB, Lau CP, Lam KSL. Prevalence, Awareness, Treatment, and Control of Hypertension Among United States Adults 1999–2004. Hypertension. 2007;49(1):69–75. 10.1161/01.HYP.0000252676.46043.18 17159087

[87] Cutler JA, Sorlie PD, Wolz M, Thom T, Fields LE, Roccella EJ. Trends in Hypertension Prevalence, Awareness, Treatment, and Control Rates in United States Adults Between 1988–1994 and 1999–2004. Hypertension. 2008;52(5):818–27. 10.1161/HYPERTENSIONAHA.108.113357 18852389

[88] Caro JJ, Speckman JL, Salas M, Raggio G, Jackson JD. Effect of initial drug choice on persistence with antihypertensive therapy: the importance of actual practice data. Canadian Medical Association Journal. 1999;160(1):41–6. 9934342 PMC1229944

[89] Mazzaglia G, Mantovani LG, Sturkenboom MCJM, Filippi A, Trifiro G, Cricelli C, et al Patterns of persistence with antihypertensive medications in newly diagnosed hypertensive patients in Italy: a retrospective cohort study in primary care. Journal of Hypertension. 2005;23(11):2093–100. 16208153 10.1097/01.hjh.0000186832.41125.8a

[90] Payne KA, Esomonde-White S. Observational studies of anti-hypertensive medication use and compliance: is drug choice a factor in treatment adherence? Curr Hypertens Rep. 2000;2(6):515–24. 11062596 10.1007/s11906-996-0035-6

[91] Rolnick SJ, Pawloski PA, Hedblom BD, Asche SE, Bruzek RJ. Patient Characteristics Associated with Medication Adherence. Clinical Medicine & Research. 2013;11(2):54–65. 23580788 10.3121/cmr.2013.1113 PMC3692389

[92] Friedman O, McAlister FA, Yun L, Campbell NRC, Tu K. Antihypertensive Drug Persistence and Compliance Among Newly Treated Elderly Hypertensives in Ontario. The American Journal of Medicine. 2010;123(2):173–81. 10.1016/j.amjmed.2009.08.008 20103027

[93] Salvi E, Wang Z, Rizzi F, Gong Y, McDonough CW, Padmanabhan S, et al Genome-Wide and Gene-Based Meta-Analyses Identify Novel Loci Influencing Blood Pressure Response to Hydrochlorothiazide. Hypertension. 2017;69(1):51–9. 10.1161/HYPERTENSIONAHA.116.08267 27802415 PMC5145728

