# Supplementary material for: Genome-wide study of resistant hypertension identified from electronic health records
Source: PLoS One. 2017 Feb 21;12(2):e0171745. doi: 10.1371/journal.pone.0171745 (PMC5319785; doi:10.1371/journal.pone.0171745)
Supplement: S3 Fig — A total of 2,530,150 SNPs were tested for an association with resistant hypertension (1,719 cases and 708 controls) among Europeans from the eMERGE I and II Network. Tests of association were performed using logistic regression assuming an additive genetic model and adjusting for sex, decade of birth, genotyping platform, median body mass index, and principal components (1–3). Each test of association was plotted where the x-axis is the chromosomal location and the y-axis is–log10 of the p-value. The most significant finding was ESR1 rs9479122 on chromosome 6, a likely false positive due to poor genotyping for this variant. (DOCX) [file pone.0171745.s003.docx]

**S3 Fig. Genome-wide association study of European Americans with resistant hypertension versus controlled hypertensives.** A total of 2,530,150 SNPs were tested for an association with resistant hypertension (1,719 cases and 708 controls) among Europeans from the eMERGE I and II Network. Tests of association were performed using logistic regression assuming an additive genetic model and adjusting for sex, decade of birth, genotyping platform, median body mass index, and principal components (1-3). Each test of association was plotted where the x-axis is the chromosomal location and the y-axis is –log_10_ of the p-value. The most significant finding was *ESR1* rs9479122 on chromosome 6, a likely false positive due to poor genotyping for this variant.

We identified this potential genotyping and imputation error after performing a look-up for *ESR1* rs9479122 in two independent datasets, INVEST [1, 2] and SPS3 [3]. INVEST was a large hypertension outcomes trial, and SPS3 was a secondary stroke prevention trial with a blood pressure arm. Both trials conducted genetic sub-studies and resistant hypertension phenotypes were constructed in a manner similar to that constructed in eMERGE. In INVEST and SPS3, *ESR1* rs9479122 is nearly monomorphic (coded allele G frequency 0.996 and 0.99937, respectively), which is in agreement with HapMap CEU estimates.

*ESR1* rs9479122 is imputed in this eMERGE dataset [4]. Prior to imputation, *ESR1* rs9479122 was genotyped directly in eMERGE I on the Illumina 660W, and allele frequencies for this SNP are available for two studies genotyped in different batches: dementia [5] and the present resistant hypertension study. The allele frequency for the dementia study samples (0.9867; n=3,335) is similar to INVEST and SPS3; however, the allele frequency of the resistant hypertension study samples genotyped on the Illumina 660W (n=1,140) is drastically different (0.5008). Examination of the pre-quality control genotyping data for eMERGE I revealed that *ESR1* rs9479122 produced genotypes in the Marshfield cataracts dataset [6] but at a call rate of 3%. No other pre-quality control eMERGE I datasets contained genotypes for *ESR1* rs9479122, suggesting that this SNP targeted by Illumina 660W did not genotype well. *ESR1* rs9479122 was targeted on Illumina BeadChips Human 370-Quad, 610-Quad, 660W-Quad, and 1M-Duo, but not on later chips.

**
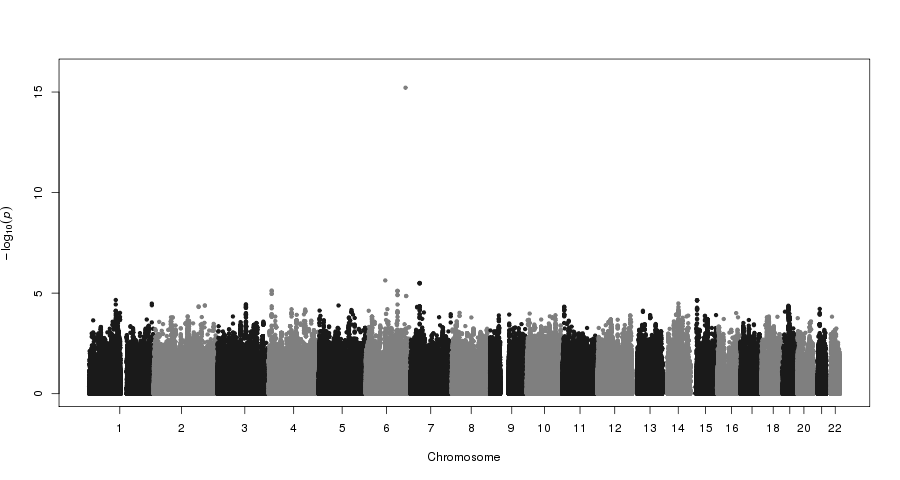
**

**References**

1. Pepine CJ, Handberg EM, Cooper-DeHoff RM, et al. A calcium antagonist vs a non–calcium antagonist hypertension treatment strategy for patients with coronary artery disease: The international verapamil-trandolapril study (invest): a randomized controlled trial. JAMA. 2003;290(21):2805-16. doi: 10.1001/jama.290.21.2805.

2. Fontana V, McDonough CW, Gong Y, El Rouby NM, Sa AC, Taylor KD, et al. Large-scale gene-centric analysis identifies polymorphisms for resistant hypertension. J Am Heart Assoc. 2014;3(6):e001398.

3. The SPSSG. Blood-pressure targets in patients with recent lacunar stroke: the SPS3 randomised trial. The Lancet. 382(9891):507-15. doi: <http://dx.doi.org/10.1016/S0140-6736(13)60852-1>.

4. Verma SS, de Andrade M, Tromp G, Kuivaniemi H, Pugh E, Namjou-Khales B, et al. Imputation and quality control steps for combining multiple genome-wide datasets. Frontiers in Genetics. 2014;5:370.

5. McDavid A, Crane PK, Newton KM, Crosslin DR, McCormick W, Weston N, et al. Enhancing the Power of Genetic Association Studies through the Use of Silver Standard Cases Derived from Electronic Medical Records. PLoS ONE. 2013;8(6):e63481.

6. Ritchie MD, Verma SS, Hall MA, Goodloe RJ, Berg RL, Carrell DS, et al. Electronic medical records and genomics (eMERGE) network exploration in cataract: several new potential susceptbility loci. Mol Vis. 2014;20:1281-95.
