# Supplementary material for: Genome-wide study of resistant hypertension identified from electronic health records
Source: PLoS One. 2017 Feb 21;12(2):e0171745. doi: 10.1371/journal.pone.0171745 (PMC5319785; doi:10.1371/journal.pone.0171745)

**S6 Fig. Genome-wide association study of European Americans with resistant hypertension versus controlled hypertensives.** A total of 2,530,150 SNPs were tested for an association with resistant hypertension (1,719 cases and 708 controls) among Europeans from the eMERGE I and II network. After removal of *ESR1* rs9479122, tests of association were performed using logistic regression assuming an additive genetic model and adjusting for sex, decade of birth, genotyping platform, median body mass index, and principal components (1-3). Each test of association was plotted on a Manhattan plot where the x-axis is the chromosomal location and the y-axis is –log_10_ of the p-value.


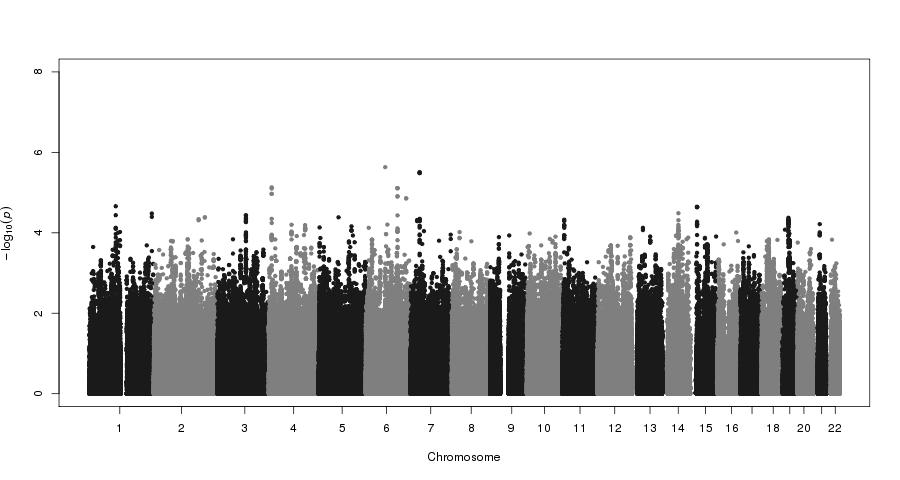

Supplement: S6 Fig — A total of 2,530,150 SNPs were tested for an association with resistant hypertension (1,719 cases and 708 controls) among Europeans from the eMERGE I and II network. After removal of ESR1 rs9479122, tests of association were performed using logistic regression assuming an additive genetic model and adjusting for sex, decade of birth, genotyping platform, median body mass index, and principal components (1–3). Each test of association was plotted on a Manhattan plot where the x-axis is the chromosomal location and the y-axis is–log10 of the p-value. (DOCX) [file pone.0171745.s006.docx]
