# Supplementary material for: Genome-wide study of resistant hypertension identified from electronic health records
Source: PLoS One. 2017 Feb 21;12(2):e0171745. doi: 10.1371/journal.pone.0171745 (PMC5319785; doi:10.1371/journal.pone.0171745)
Supplement: S1 File — (DOCX) [file pone.0171745.s008.docx]

**S1 File. electronic MEdical Records & GEnomics (eMERGE) Network Resistant Hypertension electronic health record (EHR) study inclusion and exclusion criteria.**

Subjects were determined using electronic algorithms deployed with an existing EHR.

**CASE DEFINITION**:
Subject has ≥ 4 meds simultaneous* med classes below mentioned on at least 2 occasions ≥ 1 month apart (does not have to be the same med classes in each of the 2 occasions);
OR
Subject has two outpatient (if possible) measurements of SBP > 140 or DBP > 90 at least one month after meeting medication criteria while still on 3 simultaneous med classes
AND
has 3 simultaneous* med classes below (and does not meet Case Type 1) mentioned on at least 2 occasions ≥ 1 month apart (does not have to be the same med classes in each of the 2 occasions).

*Simultaneous is defined as evidence that they are taking the medications concurrently. Such evidence could be presence of the medications in the same medication list (e.g., problem list, clinic note, or discharge summary) or via medication refill data. If using the latter method, the algorithm should find evidence of at least repeated overlapping scripts for each drug if you are using this method.

S: start
R:refill
X-axis: is time
BP: 150/80-----------145/80---154/74
Drug 1 : S----->R1------>R2---->R3---->R4
Drug 2: -----S------>absent
Drug 3:----------S------>R1---->R2---->R3--
Drug 4:-------------------S---->R1----->R2----
*At R1 of Drug 4, patient qualifies for Case Type 2*

**Note**: For those using NLP to define medications, we require a dose, strength, route, or frequency present with the medication name to insure that the medication represents a prescribed medication.

From the above, subjects with any of the following codes appearing in the record at any time were excluded:

| **ICD9 codes** | **Description** |
| --- | --- |
| 194.0 | MALIGN NEOPL ADRENAL |
| 227.0 | BENIGN NEOPLASM ADRENAL |
| 255.0, 255.1, 255.2, 255.3, 255.6, 255.8, 255.9 | Disorders of adrenal glands (excludes adrenal insufficiencies - 255.4 and 255.5) |
| 405.* | SECONDARY HYPERTENSION |
| 416.* | Chronic pulmonary heart disease |
| 581.* | Nephrotic syndrome |
| 582.* | Chronic glumerulonephritis |
| 745.* | Bulbus cordis anomalies |
| 747.1* | COARCTATION OF AORTA |

From the cases above, all subjects with the following codes were excluded only if the resistant hypertension only exists within five years before or after one of the codes below. For example, this means that one could ignore medications mentioned during a 5-year time frame before or after the below codes.

| **ICD9 codes** | **Description** |
| --- | --- |
| 242.* | Thyrotoxicosis |
| 246 | Disorder of thyrocalcitonin secretion |
| 246.8 | DISORDERS OF THYROID NEC |
| 246.9 | DISORDER OF THYROID NOS |
| 252.8 | PARATHYROID DISORDER NEC |
| 252.9 | PARATHYROID DISORDER NOS |
| 320.2 | ORGANIC SLEEP APNEA |
| 327.21 | PRIMARY CENTRAL SLEEP APNEA |
| 327.23 | OBSTRUCTIVE SLEEP APNEA |
| 327.27 | CEN SLEEP APNEA IN COND CLASS |
| 327.29 | OTHER ORGANIC SLEEP APNEA |
| 599.6* | OBSTRUCTIVE UROPATHY |

From the cases above, exclude the subject if **GFR < 30 ml/min** before the time of meeting the CASE 1 or 2 definitions or within 6 months after meeting the medication definition.

*GFR should be calculated using the Modification of Diet in Renal Disease (MDRD) formula:*
eGFR = 186 x Serum Creatinine^-1.154^ x Age^-0.203^ x [1.210 *if Black*] x [0.742 *if Female*]
*(source:* [*http://en.wikipedia.org/wiki/Renal_function#Estimated_GFR_.28eGFR.29_using_Modification_of_Diet_in_Renal_Disease_.28MDRD.29_formula*](http://en.wikipedia.org/wiki/Renal_function#Estimated_GFR_.28eGFR.29_using_Modification_of_Diet_in_Renal_Disease_.28MDRD.29_formula)*)*

Exclude all patients with an Ejection Fraction (EF or LVEF) <35% within 1 year before or after meeting the CASE 1 definition.

**CONTROL DEFINITION**:

Controls - Case 1: Subjects with controlled hypertension
Has outpatient (if possible) measurement of SBP > 140 or DBP > 90 prior to meeting medication criteria OR ICD9 401.* code at any time
AND
has 1 med from med classes below (and never has more than 1 simultaneous med class, although the med class can change)
AND
Has all SBP < 135 and DBP < 90 one month AFTER BP meds (require at least 1 BP measurement)

Controls - Case 2: Subjects without evidence of hypertension
Has no outpatient (if possible) measurement of SBP > 140 or DBP > 90
AND
No mention of any anti-hypertensive from med classes below at any time
AND
Does not have any hypertension ICD9 code: (401, 401.0, 401.9, 402.*, 403.* 404.*)

For all controls: Exclude all patients with EF < 35% or with the following ICD9 codes:

| **ICD9 codes** | **Description** |
| --- | --- |
| 194.0 | MALIGN NEOPL ADRENAL |
| 227.0 | BENIGN NEOPLASM ADRENAL |
| 255.0, 255.1, 255.2, 255.3, 255.6, 255.8, 255.9 | Disorders of adrenal glands (excludes adrenal insufficiencies - 255.4 and 255.5) |
| 405.* | SECONDARY HYPERTENSION |
| 416.* | Chronic pulmonary heart disease |
| 581.* | Nephrotic syndrome |
| 582.* | Chronic glumerulonephritis |
| 745.* | Bulbus cordis anomalies |
| 747.1* | COARCTATION OF AORTA |

Subjects with the following codes **are allowable** as controls (no matter when they occurred):

| **ICD9 codes** | **Description** |
| --- | --- |
| 242.* | Thyrotoxicosis |
| 246 | Disorder of thyrocalcitonin secretion |
| 246.8 | DISORDERS OF THYROID NEC |
| 246.9 | DISORDER OF THYROID NOS |
| 252.8 | PARATHYROID DISORDER NEC |
| 252.9 | PARATHYROID DISORDER NOS |
| 320.2 | ORGANIC SLEEP APNEA |
| 327.21 | PRIMARY CENTRAL SLEEP APNEA |
| 327.23 | OBSTRUCTIVE SLEEP APNEA |
| 327.27 | CEN SLEEP APNEA IN COND CLASS |
| 327.29 | OTHER ORGANIC SLEEP APNEA |
| 599.6* | OBSTRUCTIVE UROPATHY |

**Medication Classes**:

**Hydralazine**: Hydralazine (Apresazide, bidil, apressoline)

**Minoxidil**: (Loniten)

**Renin antagonist**: aliskiren (Tekturna)

**Central alpha agonists**: clonidine (catapres) (catapress); guanabenz; methyldopa; methyldopate

**ACEI/ARB**: candesartan (Atacand); Irbesartan (avapro); lisinopril (prinivil, zestril); trandolapril (Mavik, gopten, odrik); Losartan (cozaar); enalapril (enalaprilat); valsartan (diovan); telmisartan (Micardis); moexipril; quinapril (accupril); ramipril (altace); fosinopril (monopril); eprosartan; olmesartan (benicar); perindopril (Aceon); captopril (Capoten); benazepril (lotensin);

**Aldosterone antagonists**: spironolactone (Aldactone); eplerenone (inspra)

**Diuretics (count each instance as part of the same class, even if on more than one concurrently)**: **Thiazide**: hydrochlorothiazide (Esidrix); indapamide (lozol, natrilix); cyclothiazide; chlorothiazide; chlorthalidone; bendroflumethiazide; benzothiazide; **K-sparing diuretics**: amiloride (midamor); triamterene (Dyrenium); **Loop diuretics**: furosemide (lasix), torsemide (demadex), ethacrynic acid (ethacrynate, edecrin); bumetanide (bumex)

**Note**: the Diuretic combination meds now count as a single class: Dyazide, Moduretic, Maxzide

**Alpha antagonists**: prazosin (minipress); doxazosin (cardura)

**Non-dihydro CCBs**: verapamil (calan, covera, isoptin, verelan); diltiazem (dilt, tiazac, cardizem)

**Dihydro CCBs**: isradipine (Dynacirc); nicardipine; nifedipine (procardia); nisoldipine; felodipine (plendil); Amlodipine (norvasc, caduet); bepridil (vascor)

**Beta Blockers**: propranolol (inderal); metoprolol (toprol); labetalol (trandate); nadolol (corgard); esmolol (brevibloc); pindolol; penbutolol (levatol); Labetalol (Normodyne); atenolol (tenormin); carvedilol (coreg); bisoprolol (Zebeta);

***Thiazide/BB**: corzide , Tenoretic, lopressor HCT

***Thiazide/ACEI_ARB**: zestoretic, Avalide, hyzaar, uniretic, benicar HCT, accuretic, Teveten HCT, lotensin HCT; micardis HCT; atacand HCT; Diovan HCT; Monopril HCT

***Thiazide/aldosterone antagonist**: aldactazide

***Thiazide/Renin antagonist**: Tekturna HCT

*A match of a combination medication counts as two med classes. We have not included the generics for combination medications to avoid possible double counting of the medication classes during a search.
