# Supplementary material for: Genome-wide study of resistant hypertension identified from electronic health records
Source: PLoS One. 2017 Feb 21;12(2):e0171745. doi: 10.1371/journal.pone.0171745 (PMC5319785; doi:10.1371/journal.pone.0171745)
Supplement: S1 Table — (DOCX) [file pone.0171745.s009.docx]

**S1 Table. Medication classes considered in defining resistant hypertension status in the electronic MEdical Records & GEnomics (eMERGE) Network.**

| **Class** | **Generic names** | **Brand names** |
| --- | --- | --- |
| Angiotensin converting enzyme inhibitors (ACEI) and angiotensin receptor blockers (ARBs) | Candesartan, Irbesartan, Lisinopril, Trandolapril, Losartan, Enalapril, Valsartan, Telmisartan, Moexipril, Quinapril, Ramipril, Fosinopril, Eprosartan, Olmesarta, Perindopril, Captopril, Benazepril | Atacand, Avapro, Prinivil, Zestril, Mavik, Gopten, Odrik, Cozaar, Enalaprilat, Diovan, Micardis, Accupril, Altace, Monopril, Benicar, Aceon, Capoten, Lotensin |
| Aldosterone antagonists | Spironolactone, Eplerenone | Aldactone, Inspra |
| Alpha antagonists | Prazosin, Doxazosin | Minipress, Cardura |
| Beta blockers (BBs) | Propranolol, Metoprolol, Labetalol, Nadolol, Esmolol, Pindolol, Penbutolol, Labetalol, Atenolol, Carvedilol, Bisoprolol | Inderal, Toprol, Trandate, Corgard, Brevibloc, Levatol, Normodyne, Tenormin, Coreg, Zebeta |
| Central alpha agonists | Clonidine, Guanabenz, Methyldopa, Methyldopate | Catapres, Catapress |
| Dihydropyridine calcium channel blockers | Isradipine, Nicardipine, Nifedipine, Nisoldpine, Felodipine, Amlodipine, Bepridil | Dynacirc, Procardia, Plendil, Norvasc, Caduet, Vascor |
| Diuretics^1^(including thiazides, K-sparing diuretics, and loop diuretics) | Hydrochlorothiazide, Indapamide, Cyclothiazide, Chlorothiazide, Chlorthalidone, Bendroflumethiazide, Benzothiazide, Alioride, Triamterene, Furosemide, Torsemide, Ethacrynic acid, Bumetanide | Esidrix, Lozol, Natrilix, Midamor, Dyrenium, Lasix, Demadex, Ethacrynate, Edecrin, Bumex, Dyazide^2^, Moduretic^2^, Maxzide^2^ |
| Hydralazine | Hydralazine | Apresazide, BiDil, Apressoline |
| Minoxidil | N/A | Loniten |
| Non-dihydropyridine calcium channel blockers | Verapamil, Diltiazem | Calan, Covera, Isoptin, Verelan, Dilt, Tiazac, Cardizem |
| Renin antagonist | Aliskiren | Tekturna |
| Thiazide/ACEI_ARB^3^ | (See note^3^, below) | Zestoretic, Avalide, Hyzaar, Uniretic, Benicar HCT, Accuretic, Teveten HCT, Lotensin HCT, Micardis HCT, Atacand HCT, Diovan HCT, Monopril HCT |
| Thiazide/aldosterone antagonist^3^ | (See note^3^, below) | Aldactazide |
| Thiazide/BB^3^ | (See note^3^, below) | Corzide, Tenoretic, Lopressor HCT |
| Thiazide/renin antagonist^3^ | (See note^3^, below) | Tekturna HCT |

^1^All considered within the same class, even if the patient was prescribed more than one concurrently

^2^Diuretic combination medications were also considered one class under diuretics

^3^A patient prescribed these combination medications was considered a patient on two medication classes. Generic names were not included in the algorithm for these combination medications to avoid possible doubling counting of the medication classes.
