# Supplementary material for: Genome-wide study of resistant hypertension identified from electronic health records
Source: PLoS One. 2017 Feb 21;12(2):e0171745. doi: 10.1371/journal.pone.0171745 (PMC5319785; doi:10.1371/journal.pone.0171745)
Supplement: S2 Table — Individuals were excluded from case or control status based on ICD-9-CM codes and situations as described. In addition to exclusions based on codes, individuals were excluded from case status if there was evidence of chronic kidney disease within six months after meeting the definition for “controlled” resistant hypertension (four medication classes concurrently) or heart failure within one year before or after meeting the definition for “controlled” resistant hypertension. Chronic kidney disease was defined by an estimated glomerular filtration rate (eGFR) ≤30 ml/min, as calculated by the Modification of Diet in Renal Disease formula. Heart failure was defined as an ejection fraction (EF) or left ventricular ejection fraction (LVEF) ≤35%. Individuals with evidence of heart failure were also excluded from control status. (DOCX) [file pone.0171745.s010.docx]

**S2 Table. Exclusions from case and control definitions of resistant hypertension in the eMERGE Network.** Individuals were excluded from case or control status based on ICD-9-CM codes and situations as described. In addition to exclusions based on codes, individuals were excluded from case status if there was evidence of chronic kidney disease within six months after meeting the definition for “controlled” resistant hypertension (four medication classes concurrently) or heart failure within one year before or after meeting the definition for “controlled” resistant hypertension. Chronic kidney disease was defined by an estimated glomerular filtration rate (eGFR) ≤30 ml/min, as calculated by the Modification of Diet in Renal Disease formula. Heart failure was defined as an ejection fraction (EF) or left ventricular ejection fraction (LVEF) ≤35%. Individuals with evidence of heart failure were also excluded from control status.

| **ICD-9-CM codes** | **Description** | **Exclusion for case and control definition?** |
| --- | --- | --- |
| 194.0 | Malignant neoplasm, adrenal | Yes |
| 227.0 | Benign neoplasm, adrenal | Yes |
| 242.* | Thyrotoxicosis | Yes for cases if the resistant hypertension only exists within five years before or after this code; No for controls |
| 246 | Disorder of thyrocalcitonin secretion | Yes for cases if the resistant hypertension only exists within five years before or after this code; No for controls |
| 246.8 | Disorders of thyroid, not elsewhere classified | Yes for cases if the resistant hypertension only exists within five years before or after this code; No for controls |
| 246.9 | Disorder of thyroid, not otherwise specified | Yes for cases if the resistant hypertension only exists within five years before or after this code; No for controls |
| 252.8 | Parathyroid disorder, not elsewhere classified | Yes for cases if the resistant hypertension only exists within five years before or after this code; No for controls |
| 252.9 | Parathyroid disorder, not otherwise specified | Yes for cases if the resistant hypertension only exists within five years before or after this code; No for controls |
| 255.0, 255.1, 255.2, 255.3, 255.6, 255.8, 255.9 | Disorders of adrenal glands (excludes adrenal insufficiencies 255.4 and 255.5) | Yes |
| 320.2 | Organic sleep apnea | Yes for cases if the resistant hypertension only exists within five years before or after this code; No for controls |
| 327.21 | Primary central sleep apnea | Yes for cases if the resistant hypertension only exists within five years before or after this code; No for controls |
| 327.23 | Obstructive sleep apnea | Yes for cases if the resistant hypertension only exists within five years before or after this code; No for controls |
| 327.27 | Central sleep apnea in conditions classified elsewhere | Yes for cases if the resistant hypertension only exists within five years before or after this code; No for controls |
| 327.29 | Other organic sleep apnea | Yes for cases if the resistant hypertension only exists within five years before or after this code; No for controls |
| 405.* | Secondary hypertension | Yes |
| 416.* | Chronic pulmonary heart disease | Yes |
| 581.* | Nephrotic syndrome | Yes |
| 582.* | Chronic glomerulonephritis | Yes |
| 599.6* | Obstructive uropathy | Yes for cases if the resistant hypertension only exists within five years before or after this code; No for controls |
| 745.* | Bulbus cordis anomalies | Yes |
| 747.1* | Coarctation of aorta | Yes |
