# Supplementary material for: Genome-wide study of resistant hypertension identified from electronic health records
Source: PLoS One. 2017 Feb 21;12(2):e0171745. doi: 10.1371/journal.pone.0171745 (PMC5319785; doi:10.1371/journal.pone.0171745)
Supplement: S3 Table — Counts within parentheses represent number of additional samples identified as cases or controls but not included in the final analyses due to model convergence issues. (DOCX) [file pone.0171745.s011.docx]

**S3 Table. Resistant hypertension cases and controls for discovery, by eMERGE study site and population.** Counts within parentheses represent number of additional samples identified as cases or controls but not included in the final analyses due to model convergence issues.

| **Site** | **European Americans** | | **African Americans** | | **Hispanics** | | **Others** | |
| --- | --- | --- | --- | --- | --- | --- | --- | --- |
|  | **Resistant Hypertensives** | **Controlled Hypertensives** | **Resistant Hypertensives** | **Controlled Hypertensives** | **Resistant Hypertensives** | **Controlled Hypertensives** | **Controlled Hypertensives** | **Resistant Hypertensives** |
| Geisinger | (144) | 0 | 0 | 0 | (1) | 0 | 0 | 0 |
| Group Health | 232 | 86 | 1 | 2 | 16 | 2 | 14 | 5 |
| Marshfield | 256 | 536 | 0 | 1 | 0 | 0 | 4 | 3 |
| Mayo Clinic | 325 (24) | 27 | 1 | 0 | 1 | 0 | 7 | 2 |
| Mount Sinai | 16 | 16 | 219 | 97 | 186 | 20 | 13 | 4 |
| Northwestern | 258 | 13 | 129 | 4 | 5 | 0 | 0 | 0 |
| Vanderbilt | 692 | 37 | 447 | 18 | 3 | 0 | 12 | 3 |
| All eMERGE I and II sites | 1726 (168) | 708 | 797 | 122 | 211 (1) | 22 | 50 | 17 |
