# Supplementary material for: Genome-wide study of resistant hypertension identified from electronic health records
Source: PLoS One. 2017 Feb 21;12(2):e0171745. doi: 10.1371/journal.pone.0171745 (PMC5319785; doi:10.1371/journal.pone.0171745)
Supplement: S4 Table — The “Published GWAS” columns represent SNPs previously associated with blood pressure, systolic blood pressure, diastolic blood pressure, or hypertension among adults at genome-wide significance drawn from the NHGRI European Bioinformatics Institute (NHGRI-EBI) GWAS Catalog (http://www.ebi.ac.uk/gwas/; accessed September 2016). Published GWAS data are compared with eMERGE I and II association results for resistant hypertension among all racial/ethnic adults if the SNP is present in the dataset. Abbreviations: beta (β), p (p-value), odds ratio (OR), lower 95% confidence interval (L95), and upper 95% confidence interval (U95). Data from eMERGE I and eMERGE II are denoted by the subscripts. (DOCX) [file pone.0171745.s012.docx]

**S4 Table. Variants previously identified in GWAS of blood pressure or hypertension in current GWAS of resistant hypertension.** The “Published GWAS” columns represent SNPs previously associated with blood pressure, systolic blood pressure, diastolic blood pressure, or hypertension among adults at genome-wide significance drawn from the NHGRI European Bioinformatics Institute (NHGRI-EBI) GWAS Catalog (<http://www.ebi.ac.uk/gwas/>; accessed September 2016). Published GWAS data are compared with eMERGE I and II association results for resistant hypertension among all racial/ethnic adults if the SNP is present in the dataset. Abbreviations: beta (β), p (p-value), odds ratio (OR), lower 95% confidence interval (L95), and upper 95% confidence interval (U95). Data from eMERGE I and eMERGE II are denoted by the subscripts.

| **Published GWAS (p<5.0x10^-8^)** | | | | | | **eMERGE I and II Resistant Hypertension** | | | | | | | |
| --- | --- | --- | --- | --- | --- | --- | --- | --- | --- | --- | --- | --- | --- |
| **rs #** | **Mapped Gene** | **Published GWAS Disease Trait** | **PubMed ID** | **β** | **p** | **OR_I_** | **L95_I_** | **U95_I_** | **p_I_** | **OR_II_** | **L95_II_** | **U95_II_** | **p_II_** |
| 15285 | LPL | Triglycerides-Blood Pressure (TG-BP) | 21386085 | 0.27 | 1.00E-10 |  |  |  |  |  |  |  |  |
| 35444 | LOC102723639 - LOC101928225 | Blood pressure | 21572416 | 0.5 | 1.00E-10 | 1.3492 | 1.1370 | 1.6009 | 0.0006 | 1.34916 | 1.1370 | 1.6009 | 0.0006 |
| 133980 | LOC105372981 - MN1 | Blood pressure (smoking interaction) | 25189868 |  | 2.00E-08 |  |  |  |  |  |  |  |  |
| 133980 | LOC105372981 - MN1 | Blood pressure (smoking interaction) | 25189868 |  | 1.00E-08 |  |  |  |  |  |  |  |  |
| 198846 | HIST1H4C - HIST1H1T | Blood pressure | 21909110 | 0.478 | 2.00E-12 |  |  |  |  |  |  |  |  |
| 319690 | MAP4 | Blood pressure | 21909110 | 0.297 | 3.00E-08 | 1.0038 | 0.8380 | 1.2023 | 0.9682 | 1.00376 | 0.8380 | 1.2023 | 0.9682 |
| 381815 | PLEKHA7 | Blood pressure | 21909110 | 0.298 | 3.00E-08 | 1.0233 | 0.8481 | 1.2348 | 0.8099 | 1.02332 | 0.8481 | 1.2348 | 0.8099 |
| 381815 | PLEKHA7 | Systolic blood pressure | 19430479 | 0.65 | 2.00E-09 | 1.0233 | 0.8481 | 1.2348 | 0.8099 | 1.02332 | 0.8481 | 1.2348 | 0.8099 |
| 419076 | MECOM | Diastolic blood pressure | 21909115 | 0.241 | 2.00E-12 | 0.9670 | 0.8211 | 1.1387 | 0.6870 | 0.966955 | 0.8211 | 1.1387 | 0.6870 |
| 419076 | MECOM | Systolic blood pressure | 21909115 | 0.409 | 2.00E-13 | 0.9670 | 0.8211 | 1.1387 | 0.6870 | 0.966955 | 0.8211 | 1.1387 | 0.6870 |
| 419076 | MECOM | Blood pressure | 21909110 | 0.34 | 8.00E-13 | 0.9670 | 0.8211 | 1.1387 | 0.6870 | 0.966955 | 0.8211 | 1.1387 | 0.6870 |
| 633185 | ARHGAP42 | Diastolic blood pressure | 21909115 | 0.328 | 2.00E-15 |  |  |  |  |  |  |  |  |
| 633185 | ARHGAP42 | Systolic blood pressure | 21909115 | 0.565 | 1.00E-17 |  |  |  |  |  |  |  |  |
| 633185 | ARHGAP42 | Hypertension | 21909115 | 0.07 | 5.00E-11 |  |  |  |  |  |  |  |  |
| 633185 | ARHGAP42 | Blood pressure | 21909110 | 0.325 | 7.00E-10 |  |  |  |  |  |  |  |  |
| 653178 | ATXN2 | Blood pressure | 21909110 | 0.429 | 7.00E-20 |  |  |  |  |  |  |  |  |
| 653178 | ATXN2 | Diastolic blood pressure | 19430483 | 0.46 | 3.00E-18 |  |  |  |  |  |  |  |  |
| 780093 | GCKR | Triglycerides-Blood Pressure (TG-BP) | 21386085 | 0.18 | 3.00E-10 | 1.0109 | 0.8495 | 1.2031 | 0.9025 | 1.01094 | 0.8495 | 1.2031 | 0.9025 |
| 805303 | BAG6 | Diastolic blood pressure | 21909115 | 0.228 | 3.00E-11 |  |  |  |  |  |  |  |  |
| 805303 | BAG6 | Systolic blood pressure | 21909115 | 0.376 | 2.00E-11 |  |  |  |  |  |  |  |  |
| 805303 | BAG6 | Hypertension | 21909115 | 0.054 | 1.00E-10 |  |  |  |  |  |  |  |  |
| 820430 | SLC4A7 - LOC105377005 | Systolic blood pressure | 25249183 | 0.76 | 1.00E-12 | 1.0390 | 0.8712 | 1.2390 | 0.6705 | 1.03897 | 0.8712 | 1.2390 | 0.6705 |
| 871606 | LOC100129728 - RPL21P44 | Blood pressure | 21909110 | 0.429 | 1.00E-08 |  |  |  |  |  |  |  |  |
| 880315 | CASZ1 | Systolic blood pressure | 25249183 | 0.97 | 6.00E-10 |  |  |  |  |  |  |  |  |
| 880315 | CASZ1 | Hypertension | 25249183 |  | 2.00E-09 |  |  |  |  |  |  |  |  |
| 880315 | CASZ1 | Blood pressure | 21572416 | 0.56 | 3.00E-10 |  |  |  |  |  |  |  |  |
| 932764 | PLCE1 | Systolic blood pressure | 21909115 | 0.484 | 7.00E-16 |  |  |  |  |  |  |  |  |
| 932764 | PLCE1 | Hypertension | 21909115 | 0.055 | 9.00E-09 |  |  |  |  |  |  |  |  |
| 1004467 | CYP17A1 | Systolic blood pressure | 19430479 | 1.05 | 1.00E-10 | 1.0907 | 0.8371 | 1.4210 | 0.5205 | 1.09067 | 0.8371 | 1.4210 | 0.5205 |
| 1173766 | NPR3 - LOC340113 | Blood pressure | 21572416 | 0.63 | 2.00E-08 |  |  |  |  |  |  |  |  |
| 1173771 | NPR3 - LOC340113 | Diastolic blood pressure | 21909115 | 0.261 | 9.00E-12 |  |  |  |  |  |  |  |  |
| 1173771 | NPR3 - LOC340113 | Systolic blood pressure | 21909115 | 0.504 | 2.00E-16 |  |  |  |  |  |  |  |  |
| 1173771 | NPR3 - LOC340113 | Hypertension | 21909115 | 0.062 | 3.00E-10 |  |  |  |  |  |  |  |  |
| 1173771 | NPR3 - LOC340113 | Blood pressure | 21909110 | 0.283 | 4.00E-09 |  |  |  |  |  |  |  |  |
| 1173771 | NPR3 - LOC340113 | Blood pressure | 21909110 | 0.276 | 5.00E-09 |  |  |  |  |  |  |  |  |
| 1327235 | C20orf187 | Diastolic blood pressure | 21909115 | 0.302 | 1.00E-15 | 0.9432 | 0.7975 | 1.1155 | 0.4942 | 0.943166 | 0.7975 | 1.1155 | 0.4942 |
| 1327235 | C20orf187 | Systolic blood pressure | 21909115 | 0.34 | 2.00E-08 | 0.9432 | 0.7975 | 1.1155 | 0.4942 | 0.943166 | 0.7975 | 1.1155 | 0.4942 |
| 1327235 | C20orf187 | Blood pressure | 21909110 | 0.259 | 4.00E-08 | 0.9432 | 0.7975 | 1.1155 | 0.4942 | 0.943166 | 0.7975 | 1.1155 | 0.4942 |
| 1330225 | LOC105378885 - LOC105378887 | Blood pressure measurement (low sodium intervention) | 24165912 | 5.48 | 1.00E-08 |  |  |  |  |  |  |  |  |
| 1330225 | LOC105378885 - LOC105378887 | Blood pressure measurement (low sodium intervention) | 24165912 | 5.16 | 7.00E-09 |  |  |  |  |  |  |  |  |
| 1378942 | CSK | Diastolic blood pressure | 21909115 | 0.416 | 3.00E-26 | 1.0810 | 0.9009 | 1.2970 | 0.4021 | 1.08096 | 0.9009 | 1.2970 | 0.4021 |
| 1378942 | CSK | Systolic blood pressure | 21909115 | 0.613 | 6.00E-23 | 1.0810 | 0.9009 | 1.2970 | 0.4021 | 1.08096 | 0.9009 | 1.2970 | 0.4021 |
| 1378942 | CSK | Blood pressure | 21909110 | 0.386 | 2.00E-15 | 1.0810 | 0.9009 | 1.2970 | 0.4021 | 1.08096 | 0.9009 | 1.2970 | 0.4021 |
| 1378942 | CSK | Diastolic blood pressure | 19430483 | 0.43 | 1.00E-23 | 1.0810 | 0.9009 | 1.2970 | 0.4021 | 1.08096 | 0.9009 | 1.2970 | 0.4021 |
| 1401454 | SOX6 | Blood pressure | 23972371 | 0.45 | 5.00E-10 | 0.8425 | 0.7106 | 0.9989 | 0.0481 | 0.84252 | 0.7106 | 0.9989 | 0.0481 |
| 1446468 | FIGN - GRB14 | Blood pressure | 21909110 | 0.336 | 6.00E-12 |  |  |  |  |  |  |  |  |
| 1458038 | LOC105377304 | Diastolic blood pressure | 21909115 | 0.457 | 9.00E-25 | 0.9870 | 0.8237 | 1.1826 | 0.8871 | 0.986977 | 0.8237 | 1.1826 | 0.8871 |
| 1458038 | LOC105377304 | Systolic blood pressure | 21909115 | 0.706 | 2.00E-23 | 0.9870 | 0.8237 | 1.1826 | 0.8871 | 0.986977 | 0.8237 | 1.1826 | 0.8871 |
| 1458038 | LOC105377304 | Blood pressure | 21909110 | 0.403 | 3.00E-14 | 0.9870 | 0.8237 | 1.1826 | 0.8871 | 0.986977 | 0.8237 | 1.1826 | 0.8871 |
| 1530440 | C10orf107 | Diastolic blood pressure | 19430483 | 0.39 | 1.00E-09 | 0.8509 | 0.6878 | 1.0528 | 0.1363 | 0.850933 | 0.6878 | 1.0528 | 0.1363 |
| 1717027 | ULK4 | Blood pressure | 23972371 | 0.49 | 5.00E-13 | 0.8442 | 0.6838 | 1.0423 | 0.1148 | 0.844205 | 0.6838 | 1.0423 | 0.1148 |
| 1799945 | HFE | Diastolic blood pressure | 21909115 | 0.457 | 2.00E-15 | 0.7984 | 0.6265 | 1.0175 | 0.0676 | 0.798381 | 0.6265 | 1.0175 | 0.0676 |
| 1799945 | HFE | Systolic blood pressure | 21909115 | 0.627 | 8.00E-12 | 0.7984 | 0.6265 | 1.0175 | 0.0676 | 0.798381 | 0.6265 | 1.0175 | 0.0676 |
| 1799945 | HFE | Hypertension | 21909115 | 0.095 | 2.00E-10 | 0.7984 | 0.6265 | 1.0175 | 0.0676 | 0.798381 | 0.6265 | 1.0175 | 0.0676 |
| 1887320 | C20orf187 | Systolic blood pressure | 25249183 | 0.78 | 1.00E-08 | 0.9387 | 0.7935 | 1.1104 | 0.4605 | 0.938711 | 0.7935 | 1.1104 | 0.4605 |
| 1887320 | C20orf187 | Diastolic blood pressure | 25249183 | 0.43 | 2.00E-08 | 0.9387 | 0.7935 | 1.1104 | 0.4605 | 0.938711 | 0.7935 | 1.1104 | 0.4605 |
| 1902859 | LOC105377304 | Systolic blood pressure | 25249183 | 1.34 | 2.00E-22 |  |  |  |  |  |  |  |  |
| 1902859 | LOC105377304 | Diastolic blood pressure | 25249183 | 0.71 | 4.00E-20 |  |  |  |  |  |  |  |  |
| 1902859 | LOC105377304 | Hypertension | 25249183 |  | 8.00E-18 |  |  |  |  |  |  |  |  |
| 2021783 | TNXB | Systolic blood pressure | 25249183 | 0.68 | 3.00E-09 |  |  |  |  |  |  |  |  |
| 2021783 | TNXB | Diastolic blood pressure | 25249183 | 0.49 | 2.00E-12 |  |  |  |  |  |  |  |  |
| 2021783 | TNXB | Hypertension | 25249183 |  | 4.00E-11 |  |  |  |  |  |  |  |  |
| 2030114 | LOC102723323 - HNRNPA1P48 | Blood pressure measurement (high sodium and potassium intervention) | 24165912 | 3.14 | 7.00E-13 |  |  |  |  |  |  |  |  |
| 2071518 | NOV | Blood pressure | 21909110 | 0.312 | 4.00E-09 | 0.8835 | 0.7332 | 1.0647 | 0.1927 | 0.883546 | 0.7332 | 1.0647 | 0.1927 |
| 2226284 | LRRC7 | Hypertension risk in short sleep duration | 22322875 | 5.91 | 3.00E-08 | 1.0626 | 0.9010 | 1.2531 | 0.4708 | 1.06256 | 0.9010 | 1.2531 | 0.4708 |
| 2266788 | APOA5 | Triglycerides-Blood Pressure (TG-BP) | 21386085 | 0.37 | 4.00E-08 |  |  |  |  |  |  |  |  |
| 2268365 | LRP2 | Blood pressure (smoking interaction) | 25189868 |  | 5.00E-08 | 1.0640 | 0.8246 | 1.3729 | 0.6336 | 1.06396 | 0.8246 | 1.3729 | 0.6336 |
| 2384550 | LOC105369999 - LOC102723639 | Diastolic blood pressure | 19430479 | 0.35 | 4.00E-08 | 1.2550 | 1.0506 | 1.4991 | 0.0121 | 1.255 | 1.0506 | 1.4991 | 0.0121 |
| 2521501 | FES | Diastolic blood pressure | 21909115 | 0.359 | 2.00E-15 |  |  |  |  |  |  |  |  |
| 2521501 | FES | Systolic blood pressure | 21909115 | 0.65 | 5.00E-19 |  |  |  |  |  |  |  |  |
| 2521501 | FES | Blood pressure | 21909110 | 0.344 | 3.00E-08 |  |  |  |  |  |  |  |  |
| 2681472 | ATP2B1 | Diastolic blood pressure | 19430479 | 0.5 | 1.00E-09 | 1.1567 | 0.9198 | 1.4547 | 0.2132 | 1.15674 | 0.9198 | 1.4547 | 0.2132 |
| 2681472 | ATP2B1 | Hypertension | 19430479 | 0.15 | 2.00E-11 | 1.1567 | 0.9198 | 1.4547 | 0.2132 | 1.15674 | 0.9198 | 1.4547 | 0.2132 |
| 2681492 | ATP2B1 | Systolic blood pressure | 19430479 | 0.85 | 4.00E-11 | 1.2040 | 0.9589 | 1.5117 | 0.1099 | 1.204 | 0.9589 | 1.5117 | 0.1099 |
| 2782980 | LOC105378492 | Blood pressure | 21909110 | 0.338 | 2.00E-09 |  |  |  |  |  |  |  |  |
| 2932538 | CAPZA1 - MOV10 | Diastolic blood pressure | 21909115 | 0.24 | 1.00E-09 | 0.8849 | 0.7333 | 1.0680 | 0.2016 | 0.884935 | 0.7333 | 1.0680 | 0.2016 |
| 2932538 | CAPZA1 - MOV10 | Systolic blood pressure | 21909115 | 0.388 | 1.00E-09 | 0.8849 | 0.7333 | 1.0680 | 0.2016 | 0.884935 | 0.7333 | 1.0680 | 0.2016 |
| 2954033 | LOC105375745 | Triglycerides-Blood Pressure (TG-BP) | 21386085 | 0.17 | 9.00E-09 |  |  |  |  |  |  |  |  |
| 3096277 | CDH13 | Blood pressure | 17903302 |  | 1.00E-09 | 0.9560 | 0.7806 | 1.1708 | 0.6633 | 0.955988 | 0.7806 | 1.1708 | 0.6633 |
| 3184504 | SH2B3 | Diastolic blood pressure | 21909115 | 0.448 | 4.00E-25 | 0.9453 | 0.7977 | 1.1203 | 0.5164 | 0.945343 | 0.7977 | 1.1203 | 0.5164 |
| 3184504 | SH2B3 | Systolic blood pressure | 19430479 | 0.58 | 5.00E-09 | 0.9453 | 0.7977 | 1.1203 | 0.5164 | 0.945343 | 0.7977 | 1.1203 | 0.5164 |
| 3184504 | SH2B3 | Diastolic blood pressure | 19430479 | 0.48 | 3.00E-14 | 0.9453 | 0.7977 | 1.1203 | 0.5164 | 0.945343 | 0.7977 | 1.1203 | 0.5164 |
| 4373814 | SLC39A12 - CACNB2 | Systolic blood pressure | 21909115 | 0.373 | 5.00E-11 |  |  |  |  |  |  |  |  |
| 4373814 | SLC39A12 - CACNB2 | Diastolic blood pressure | 21909115 | 0.218 | 4.00E-10 |  |  |  |  |  |  |  |  |
| 4409766 | C10orf32-ASMT, C10orf32 | Systolic blood pressure | 25249183 | 1.24 | 6.00E-17 | 1.0588 | 0.8128 | 1.3792 | 0.6722 | 1.05878 | 0.8128 | 1.3792 | 0.6722 |
| 4409766 | C10orf32-ASMT, C10orf32 | Diastolic blood pressure | 25249183 | 0.59 | 6.00E-13 | 1.0588 | 0.8128 | 1.3792 | 0.6722 | 1.05878 | 0.8128 | 1.3792 | 0.6722 |
| 4409766 | C10orf32-ASMT, C10orf32 | Hypertension | 25249183 |  | 7.00E-13 | 1.0588 | 0.8128 | 1.3792 | 0.6722 | 1.05878 | 0.8128 | 1.3792 | 0.6722 |
| 4573996 | LOC105372222 - LOC105372223 | Blood pressure (smoking interaction) | 25189868 |  | 9.00E-09 |  |  |  |  |  |  |  |  |
| 4590817 | C10orf107 | Blood pressure | 21909110 | 0.576 | 2.00E-18 | 1.0627 | 0.8459 | 1.3350 | 0.6017 | 1.06266 | 0.8459 | 1.3350 | 0.6017 |
| 4757391 | SOX6 | Systolic blood pressure | 25249183 | 0.88 | 5.00E-09 | 0.7467 | 0.6063 | 0.9196 | 0.0057 | 0.74666 | 0.6063 | 0.9196 | 0.0057 |
| 4757391 | SOX6 | Diastolic blood pressure | 25249183 | 0.49 | 5.00E-09 | 0.7467 | 0.6063 | 0.9196 | 0.0057 | 0.74666 | 0.6063 | 0.9196 | 0.0057 |
| 4842666 | LOC102724782 | Blood pressure | 24954895 | 0.71 | 4.00E-08 |  |  |  |  |  |  |  |  |
| 6015450 | ZNF831 | Diastolic blood pressure | 21909115 | 0.557 | 6.00E-23 |  |  |  |  |  |  |  |  |
| 6015450 | ZNF831 | Systolic blood pressure | 21909115 | 0.896 | 4.00E-23 |  |  |  |  |  |  |  |  |
| 6015450 | ZNF831 | Hypertension | 21909115 | 0.11 | 4.00E-14 |  |  |  |  |  |  |  |  |
| 6015450 | ZNF831 | Blood pressure | 21909110 | 0.521 | 2.00E-12 |  |  |  |  |  |  |  |  |
| 6495122 | CPLX3 - ULK3 | Diastolic blood pressure | 19430479 | 0.4 | 2.00E-10 |  |  |  |  |  |  |  |  |
| 6736587 | LOC102724542 | Orthostatic hypotension | 24124408 | 1.38 | 5.00E-08 | 0.9096 | 0.7607 | 1.0878 | 0.2990 | 0.909639 | 0.7607 | 1.0878 | 0.2990 |
| 6825911 | LOC105377362 - ENPEP | Blood pressure | 21572416 | 0.39 | 9.00E-09 |  |  |  |  |  |  |  |  |
| 7129220 | CAND1.11 | Systolic blood pressure | 21909115 | 0.619 | 3.00E-12 | 0.8019 | 0.6090 | 1.0560 | 0.1148 | 0.80191 | 0.6090 | 1.0560 | 0.1148 |
| 7577262 | MSL3P1 - TRPM8 | Blood pressure measurement (cold pressor test) | 24165912 | 1.71 | 3.00E-08 |  |  |  |  |  |  |  |  |
| 7638110 | BPESC1 - PISRT1 | Obesity-related traits | 22013104 |  | 5.00E-08 | 1.1787 | 0.8740 | 1.5898 | 0.2830 | 1.17874 | 0.8740 | 1.5898 | 0.2830 |
| 7801190 | SLC12A9 | Hypertension | 21347282 | 1.31 | 3.00E-08 |  |  |  |  |  |  |  |  |
| 8002688 | PIBF1 | Blood pressure measurement (low sodium intervention) | 24165912 | 2.04 | 2.00E-09 | 0.8650 | 0.5290 | 1.4144 | 0.5617 | 0.865012 | 0.5290 | 1.4144 | 0.5617 |
| 9313772 | LOC101927697 | Blood pressure | 21909110 | 0.335 | 1.00E-11 |  |  |  |  |  |  |  |  |
| 9318552 | RNF219-AS1 | Diastolic blood pressure (alcohol consumption interaction) | 24376456 | 0.389 | 5.00E-08 | 1.0526 | 0.8710 | 1.2722 | 0.5957 | 1.05263 | 0.8710 | 1.2722 | 0.5957 |
| 9663362 | PLCE1 | Blood pressure | 21909110 | 0.271 | 5.00E-09 |  |  |  |  |  |  |  |  |
| 9810888 | CACNA1D | Systolic blood pressure | 25249183 | 0.53 | 5.00E-08 |  |  |  |  |  |  |  |  |
| 9810888 | CACNA1D | Diastolic blood pressure | 25249183 | 0.39 | 4.00E-12 |  |  |  |  |  |  |  |  |
| 9815354 | ULK4 | Diastolic blood pressure | 19430479 | 0.49 | 3.00E-09 | 0.8398 | 0.6771 | 1.0417 | 0.1113 | 0.839849 | 0.6771 | 1.0417 | 0.1113 |
| 10745332 | CAPZA1 | Systolic blood pressure | 25249183 | 0.96 | 3.00E-09 | 0.8871 | 0.7353 | 1.0703 | 0.2103 | 0.887148 | 0.7353 | 1.0703 | 0.2103 |
| 10745332 | CAPZA1 | Hypertension | 25249183 |  | 3.00E-09 | 0.8871 | 0.7353 | 1.0703 | 0.2103 | 0.887148 | 0.7353 | 1.0703 | 0.2103 |
| 10826334 | LOC105378318 - LOC105378319 | Systolic blood pressure (alcohol consumption interaction) | 24376456 | 0.238 | 1.00E-08 | 1.0528 | 0.7541 | 1.4697 | 0.7628 | 1.05279 | 0.7541 | 1.4697 | 0.7628 |
| 10826334 | LOC105378318 - LOC105378319 | Systolic blood pressure (alcohol consumption interaction) | 24376456 | 0.864 | 4.00E-08 | 1.0528 | 0.7541 | 1.4697 | 0.7628 | 1.05279 | 0.7541 | 1.4697 | 0.7628 |
| 10930597 | LOC105373744 - LOC643997 | Blood pressure measurement (high sodium and potassium intervention) | 24165912 | 3.2 | 4.00E-08 |  |  |  |  |  |  |  |  |
| 10930597 | LOC105373744 - LOC643997 | Blood pressure measurement (high sodium and potassium intervention) | 24165912 | 3.48 | 1.00E-08 |  |  |  |  |  |  |  |  |
| 10930597 | LOC105373744 - LOC643997 | Blood pressure measurement (low sodium intervention) | 24165912 | 3.37 | 4.00E-08 |  |  |  |  |  |  |  |  |
| 11014166 | CACNB2 | Diastolic blood pressure | 19430479 | 0.37 | 1.00E-08 |  |  |  |  |  |  |  |  |
| 11066280 | HECTD4 | Diastolic blood pressure | 25249183 | 0.62 | 3.00E-10 |  |  |  |  |  |  |  |  |
| 11066280 | HECTD4 | Blood pressure | 21572416 | 1.56 | 8.00E-31 |  |  |  |  |  |  |  |  |
| 11066280 | HECTD4 | Blood pressure | 21572416 | 1.01 | 1.00E-35 |  |  |  |  |  |  |  |  |
| 11067763 | LOC105370003 | Systolic blood pressure | 25249183 | 0.81 | 6.00E-16 |  |  |  |  |  |  |  |  |
| 11067763 | LOC105370003 | Diastolic blood pressure | 25249183 | 0.51 | 2.00E-18 |  |  |  |  |  |  |  |  |
| 11099098 | LOC105377304 | Blood pressure | 24954895 | 0.97 | 2.00E-11 |  |  |  |  |  |  |  |  |
| 11191548 | CNNM2 - NT5C2 | Systolic blood pressure | 21909115 | 1.095 | 7.00E-26 | 1.2449 | 0.9301 | 1.6662 | 0.1406 | 1.24491 | 0.9301 | 1.6662 | 0.1406 |
| 11191548 | CNNM2 - NT5C2 | Blood pressure | 21909110 | 0.529 | 8.00E-11 | 1.2449 | 0.9301 | 1.6662 | 0.1406 | 1.24491 | 0.9301 | 1.6662 | 0.1406 |
| 11191548 | CNNM2 - NT5C2 | Blood pressure | 21572416 | 1.18 | 4.00E-17 | 1.2449 | 0.9301 | 1.6662 | 0.1406 | 1.24491 | 0.9301 | 1.6662 | 0.1406 |
| 11191548 | CNNM2 - NT5C2 | Blood pressure | 21572416 | 0.58 | 7.00E-12 | 1.2449 | 0.9301 | 1.6662 | 0.1406 | 1.24491 | 0.9301 | 1.6662 | 0.1406 |
| 11191548 | CNNM2 - NT5C2 | Systolic blood pressure | 19430483 | 1.16 | 7.00E-24 | 1.2449 | 0.9301 | 1.6662 | 0.1406 | 1.24491 | 0.9301 | 1.6662 | 0.1406 |
| 11191593 | NT5C2 | Blood pressure | 21909110 | 0.66 | 1.00E-15 |  |  |  |  |  |  |  |  |
| 11222084 | LOC646383 | Blood pressure | 21909110 | 0.337 | 2.00E-11 |  |  |  |  |  |  |  |  |
| 11823543 | LOC105369514 - ZPR1 | Triglycerides-Blood Pressure (TG-BP) | 21386085 | 0.35 | 3.00E-09 |  |  |  |  |  |  |  |  |
| 11825181 | BUD13 | Triglycerides-Blood Pressure (TG-BP) | 21386085 | 0.32 | 3.00E-09 | 0.8083 | 0.5873 | 1.1126 | 0.1889 | 0.808333 | 0.5873 | 1.1126 | 0.1889 |
| 11887188 | LOC105373933 - LOC105373935 | Blood pressure measurement (high sodium and potassium intervention) | 24165912 | 3.28 | 2.00E-08 |  |  |  |  |  |  |  |  |
| 11953630 | LOC101927697 - LOC105377681 | Diastolic blood pressure | 21909115 | 0.281 | 4.00E-13 |  |  |  |  |  |  |  |  |
| 11953630 | LOC101927697 - LOC105377681 | Systolic blood pressure | 21909115 | 0.412 | 3.00E-11 |  |  |  |  |  |  |  |  |
| 12149862 | CYB5B | Blood pressure (smoking interaction) | 25189868 |  | 4.00E-09 |  |  |  |  |  |  |  |  |
| 12258967 | CACNB2 | Blood pressure | 21909110 | 0.431 | 2.00E-16 |  |  |  |  |  |  |  |  |
| 12416687 | LOC105378462, C10orf32-ASMT | Blood pressure | 24954895 | 0.59 | 4.00E-09 | 0.9373 | 0.7713 | 1.1390 | 0.5145 | 0.93727 | 0.7713 | 1.1390 | 0.5145 |
| 12946454 | PLCD3 | Systolic blood pressure | 19430483 | 0.57 | 1.00E-08 |  |  |  |  |  |  |  |  |
| 13002573 | FIGN - GRB14 | Blood pressure | 21909110 | 0.31 | 2.00E-08 | 0.9227 | 0.7588 | 1.1220 | 0.4198 | 0.922691 | 0.7588 | 1.1220 | 0.4198 |
| 13082711 | SLC4A7 - LOC105377005 | Diastolic blood pressure | 21909115 | 0.238 | 4.00E-09 |  |  |  |  |  |  |  |  |
| 13082711 | SLC4A7 - LOC105377005 | Blood pressure | 21909110 | 0.336 | 5.00E-09 |  |  |  |  |  |  |  |  |
| 13107325 | SLC39A8 | Diastolic blood pressure | 21909115 | 0.684 | 2.00E-17 | 1.2343 | 0.8913 | 1.7092 | 0.2059 | 1.23427 | 0.8913 | 1.7092 | 0.2059 |
| 13107325 | SLC39A8 | Systolic blood pressure | 21909115 | 0.981 | 3.00E-14 | 1.2343 | 0.8913 | 1.7092 | 0.2059 | 1.23427 | 0.8913 | 1.7092 | 0.2059 |
| 13107325 | SLC39A8 | Blood pressure | 21909110 | 0.633 | 1.00E-10 | 1.2343 | 0.8913 | 1.7092 | 0.2059 | 1.23427 | 0.8913 | 1.7092 | 0.2059 |
| 13139571 | GUCY1A3, LOC105377506 | Diastolic blood pressure | 21909115 | 0.26 | 2.00E-10 |  |  |  |  |  |  |  |  |
| 13143871 | GUCY1A3 | Systolic blood pressure | 25249183 | 0.96 | 5.00E-08 | 1.0390 | 0.8600 | 1.2553 | 0.6917 | 1.039 | 0.8600 | 1.2553 | 0.6917 |
| 13209747 | LOC105377992 - LOC105377991 | Blood pressure | 23972371 | 0.85 | 3.00E-10 | 0.9707 | 0.8199 | 1.1493 | 0.7301 | 0.97072 | 0.8199 | 1.1493 | 0.7301 |
| 13209747 | LOC105377992 - LOC105377991 | Blood pressure | 23972371 | 0.56 | 2.00E-11 | 0.9707 | 0.8199 | 1.1493 | 0.7301 | 0.97072 | 0.8199 | 1.1493 | 0.7301 |
| 13333226 | UMOD | Hypertension | 21082022 | 1.15 | 4.00E-11 |  |  |  |  |  |  |  |  |
| 13390641 | LOC105373519 - LOC105373520 | Blood pressure (anthropometric measures interaction) | 24903457 | 0.59 | 5.00E-08 | 1.1052 | 0.8245 | 1.4813 | 0.5038 | 1.10515 | 0.8245 | 1.4813 | 0.5038 |
| 16833934 | LOC730129 - LOC102724419 | Blood pressure | 24954895 | 1.63 | 1.00E-08 |  |  |  |  |  |  |  |  |
| 16849225 | FIGN - GRB14 | Blood pressure | 21572416 | 0.75 | 4.00E-11 | 0.9031 | 0.7428 | 1.0979 | 0.3058 | 0.903069 | 0.7428 | 1.0979 | 0.3058 |
| 16890334 | LOC105377865 - IRAK1BP1 | Blood pressure measurement (high sodium and potassium intervention) | 24165912 | 5.38 | 1.00E-10 |  |  |  |  |  |  |  |  |
| 16890334 | LOC105377865 - IRAK1BP1 | Blood pressure measurement (high sodium intervention) | 24165912 | 5.43 | 4.00E-09 |  |  |  |  |  |  |  |  |
| 16933812 | PAX5 | Obesity-related traits | 22013104 |  | 9.00E-09 | 0.8933 | 0.7483 | 1.0664 | 0.2114 | 0.893291 | 0.7483 | 1.0664 | 0.2114 |
| 16948048 | LOC102724596 | Diastolic blood pressure | 19430483 | 0.31 | 5.00E-09 |  |  |  |  |  |  |  |  |
| 16998073 | LOC105377304 - FGF5 | Diastolic blood pressure | 19430483 | 0.5 | 1.00E-21 |  |  |  |  |  |  |  |  |
| 17030613 | CAPZA1 | Blood pressure | 21572416 | 0.38 | 1.00E-08 | 0.9091 | 0.7372 | 1.1210 | 0.3719 | 0.909065 | 0.7372 | 1.1210 | 0.3719 |
| 17080102 | PLEKHG1 | Blood pressure | 23972371 | 1.02 | 5.00E-08 | 1.2164 | 0.8594 | 1.7217 | 0.2709 | 1.21638 | 0.8594 | 1.7217 | 0.2709 |
| 17080102 | PLEKHG1 | Blood pressure | 23972371 | 0.74 | 2.00E-11 | 1.2164 | 0.8594 | 1.7217 | 0.2709 | 1.21638 | 0.8594 | 1.7217 | 0.2709 |
| 17135875 | FAM185A, FBXL13 | Blood pressure measurement (cold pressor test) | 24165912 | 3.44 | 4.00E-09 |  |  |  |  |  |  |  |  |
| 17249754 | ATP2B1 | Systolic blood pressure | 25249183 | 1.03 | 4.00E-12 |  |  |  |  |  |  |  |  |
| 17249754 | ATP2B1 | Diastolic blood pressure | 25249183 | 0.52 | 2.00E-10 |  |  |  |  |  |  |  |  |
| 17249754 | ATP2B1 | Hypertension | 25249183 |  | 2.00E-08 |  |  |  |  |  |  |  |  |
| 17249754 | ATP2B1 | Blood pressure | 24001895 | 0.8 | 7.00E-15 |  |  |  |  |  |  |  |  |
| 17249754 | ATP2B1 | Blood pressure | 21909110 | 0.557 | 1.00E-17 |  |  |  |  |  |  |  |  |
| 17249754 | ATP2B1 | Blood pressure | 21909110 | 0.392 | 6.00E-10 |  |  |  |  |  |  |  |  |
| 17249754 | ATP2B1 | Blood pressure | 21572416 | 1.17 | 8.00E-20 |  |  |  |  |  |  |  |  |
| 17249754 | ATP2B1 | Blood pressure | 21572416 | 0.58 | 2.00E-13 |  |  |  |  |  |  |  |  |
| 17367504 | MTHFR | Blood pressure | 21909110 | 0.534 | 2.00E-16 | 1.0110 | 0.8073 | 1.2663 | 0.9238 | 1.01104 | 0.8073 | 1.2663 | 0.9238 |
| 17367504 | MTHFR | Systolic blood pressure | 19430483 | 0.85 | 2.00E-13 | 1.0110 | 0.8073 | 1.2663 | 0.9238 | 1.01104 | 0.8073 | 1.2663 | 0.9238 |
| 17428471 | RPL35P4 - LOC105375206 | Blood pressure | 23972371 | 1.2 | 2.00E-12 | 0.7221 | 0.5302 | 0.9833 | 0.0371 | 0.722074 | 0.5302 | 0.9833 | 0.0371 |
| 17428471 | RPL35P4 - LOC105375206 | Blood pressure | 23972371 | 0.61 | 2.00E-09 | 0.7221 | 0.5302 | 0.9833 | 0.0371 | 0.722074 | 0.5302 | 0.9833 | 0.0371 |
| 17477177 | CCDC71L - PIK3CG | Blood pressure | 21909110 | 0.418 | 2.00E-13 | 0.8955 | 0.7233 | 1.1087 | 0.3104 | 0.895496 | 0.7233 | 1.1087 | 0.3104 |
| 17608766 | GOSR2 | Systolic blood pressure | 21909115 | 0.556 | 1.00E-10 | 0.9927 | 0.7699 | 1.2799 | 0.9551 | 0.992659 | 0.7699 | 1.2799 | 0.9551 |
| 17608766 | GOSR2 | Blood pressure | 21909110 | 0.534 | 6.00E-15 | 0.9927 | 0.7699 | 1.2799 | 0.9551 | 0.992659 | 0.7699 | 1.2799 | 0.9551 |
|  | GPR39; GPR39 | Hypertension | 21626137 |  | 1.00E-10 |  |  |  |  |  |  |  |  |
|  | LOC105374725 - SLC1A3; LOC105374725 - SLC1A3 | Hypertension | 21626137 |  | 5.00E-13 |  |  |  |  |  |  |  |  |
|  | MYO6; MYO6 | Hypertension | 21626137 |  | 3.00E-10 |  |  |  |  |  |  |  |  |
|  | MIR548AZ - NOV; MIR548AZ - NOV | Hypertension | 21626137 |  | 3.00E-16 |  |  |  |  |  |  |  |  |
|  | ZFAT; ZFAT | Hypertension | 21626137 |  | 2.00E-44 |  |  |  |  |  |  |  |  |
|  | MACROD2; MACROD2 | Hypertension | 21626137 |  | 7.00E-09 |  |  |  |  |  |  |  |  |
|  | LOC105374832 - LOC105374833; LOC105374832 - LOC105374833 | Hypertension | 21626137 |  | 2.00E-09 |  |  |  |  |  |  |  |  |
